# Iqcg Is Essential for Sperm Flagellum Formation in Mice

**DOI:** 10.1371/journal.pone.0098053

**Published:** 2014-05-21

**Authors:** Ren-Ke Li, Jue-Ling Tan, Li-Ting Chen, Jing-Sheng Feng, Wen-Xue Liang, Xue-Jiang Guo, Ping Liu, Zhu Chen, Jia-Hao Sha, Yi-Fei Wang, Sai-Juan Chen

**Affiliations:** 1 State Key Laboratory of Medical Genomics, Institute of Health Sciences, Shanghai Institutes for Biological Sciences and Graduate School, Chinese Academy of Sciences and Shanghai Institute of Hematology, Rui Jin Hospital affiliated to Shanghai Jiao Tong University School of Medicine, Shanghai, China; 2 Department of Histology and Embryology, School of Medicine, Shanghai Jiao Tong University, Shanghai, China; 3 State Key Laboratory of Reproductive Medicine, Department of Histology and Embryology, Nanjing Medical University, Nanjing, Jiangsu Province, China; 4 Central Laboratory, Lianyungang First People's Hospital, Lianyungang, China; Universidad Nacional Autónoma de México, Mexico

## Abstract

Mammalian spermatogenesis comprises three successive phases: mitosis phase, meiosis phase, and spermiogenesis. During spermiogenesis, round spermatid undergoes dramatic morphogenesis to give rise to mature spermatozoon, including the condensation and elongation of nucleus, development of acrosome, formation of flagellum, and removal of excessive cytoplasm. Although these transformations are well defined at the morphological level, the mechanisms underlying these intricate processes are largely unknown. Here, we report that *Iqcg*, which was previously characterized to be involved in a chromosome translocation of human leukemia, is highly expressed in the spermatogenesis of mice and localized to the manchette in developing spermatids. *Iqcg* knockout causes male infertility, due to severe defects of spermiogenesis and resultant total immobility of spermatozoa. The axoneme in the *Iqc*g knockout sperm flagellum is disorganized and hardly any typical (“9+2”) pattern of microtubule arrangement could be found in *Iqcg* knockout spermatids. Iqcg interacts with calmodulin in a calcium dependent manner in the testis, suggesting that Iqcg may play a role through calcium signaling. Furthermore, cilia structures in the trachea and oviduct, as well as histological appearances of other major tissues, remain unchanged in the *Iqcg* knockout mice, suggesting that *Iqcg* is specifically required for spermiogenesis in mammals. These results might also provide new insights into the genetic causes of human infertility.

## Introduction

Mammalian spermatogenesis is a complex and tightly controlled process occurring in the seminiferous tubule of testis [1]. Generally, it can be divided into three consecutive phases: mitotic phase, meiotic phase, and spermiogenesis. In the mitotic phase, spermatogonia undergo serial mitotic divisions and give rise to spermatocytes. Then the round haploid spermatids are generated by two successive meiotic divisions of spermatocytes. The last phase, spermiogenesis, refers to the dramatic morphogenesis of the round spermatids to differentiate into the tadpole-like spermatozoa, which includes the condensation and elongation of nucleus, development of acrosome, formation of flagellum and disposal of excessive cytoplasm. These spermatozoa will go through the tract of epididymis to obtain further maturation and eventually become motile and functional spermatozoa which can fertilize oocytes.

Although the morphological changes during spermiogenesis were well defined in various species [2], [3], [4], the mechanisms underlying these processes are largely unknown. During the last two decades, the development of gene targeting technique in mice helped researchers to identify plenty of genes that are critical for normal spermiogenesis [1], [5]. Among them are genes essential for nuclear condensation and head shaping (e.g., *Hook1*, *Prm1*, *Prm2*, *Tnp1*, and *Tnp2*) [6], [7], [8], [9], [10], acrosome development (e.g., *Hrb*, *Gopc*, and *Csnk2a2*) [11], [12], [13] and flagellum formation (e.g., *Bbs2*, *Tektin-t*, *Akap4*, *Meig1*, and *Pacrg*) [14], [15], [16], [17], [18]. Although some of these gene products were demonstrated to bind each other and function in a cooperative manner [17], [19], the exact molecular roles and network of these proteins are still elusive and need to be further investigated.

Human IQ motif containing G (*IQCG*) gene was first reported to be involved in the chromosome translocation in a case of acute T-lymphoid/myeloid leukemia [20]. In that case, the gene *IQCG* on chromosome 3 was disrupted, and its C-terminal was fused to the N-terminal of the gene *Nucleoporin 98* (*NUP98*) on chromosome 11, resulting in the formation of a chimeric protein NUP98-IQCG. NUP98-IQCG displayed oncogenic properties, which could block both the differentiation and apoptosis of myeloid cells *in vitro* [20] and initiate an acute myeloid leukemia phenotype in the murine bone marrow transplantation model *in vivo* (unpublished data), suggesting its critical role in leukemogenesis. However, the physiological role of the wild-type (WT) *IQCG* in mammals represented a challenge. In the present work, we find the expression of *Iqcg* is prominently enriched in the testis of mice and reveals an ordered expression pattern during spermatogenesis. *Iqcg* knockout (KO) mice are sterile, due to the severe malformation and total immobility of their spermatozoa. The axoneme in the KO sperm flagellum is disorganized and hardly any typical normal “9+2” pattern, composed of two central microtubules surrounded by nine microtubular doublets, could be found in the KO spermatids. However, the cilia structures in the trachea and oviduct, as well as histological appearances of other major tissues, remain unaffected in the KO mice, suggesting *Iqcg*'s specific role in spermiogenesis.

It is worth pointing out that during our manuscript preparation, another group also reported similar sperm phenotypes using both *Iqcg*-mutagenesis and KO strategies [21]. However, our report here performed more detailed analyses of *Iqcg* KO spermiogenesis, as well as the histology of other tissues after *Iqcg* KO, especially for these tissues with motile cilia, e.g., trachea and oviduct. Furthermore, we also experimentally identified the interaction between Iqcg and calmodulin, thus providing more clues to the molecular function of Iqcg in spermiogenesis.

## Results

### Iqcg was highly and orderly expressed in the spermatogenesis of mice

**Figure 1:**
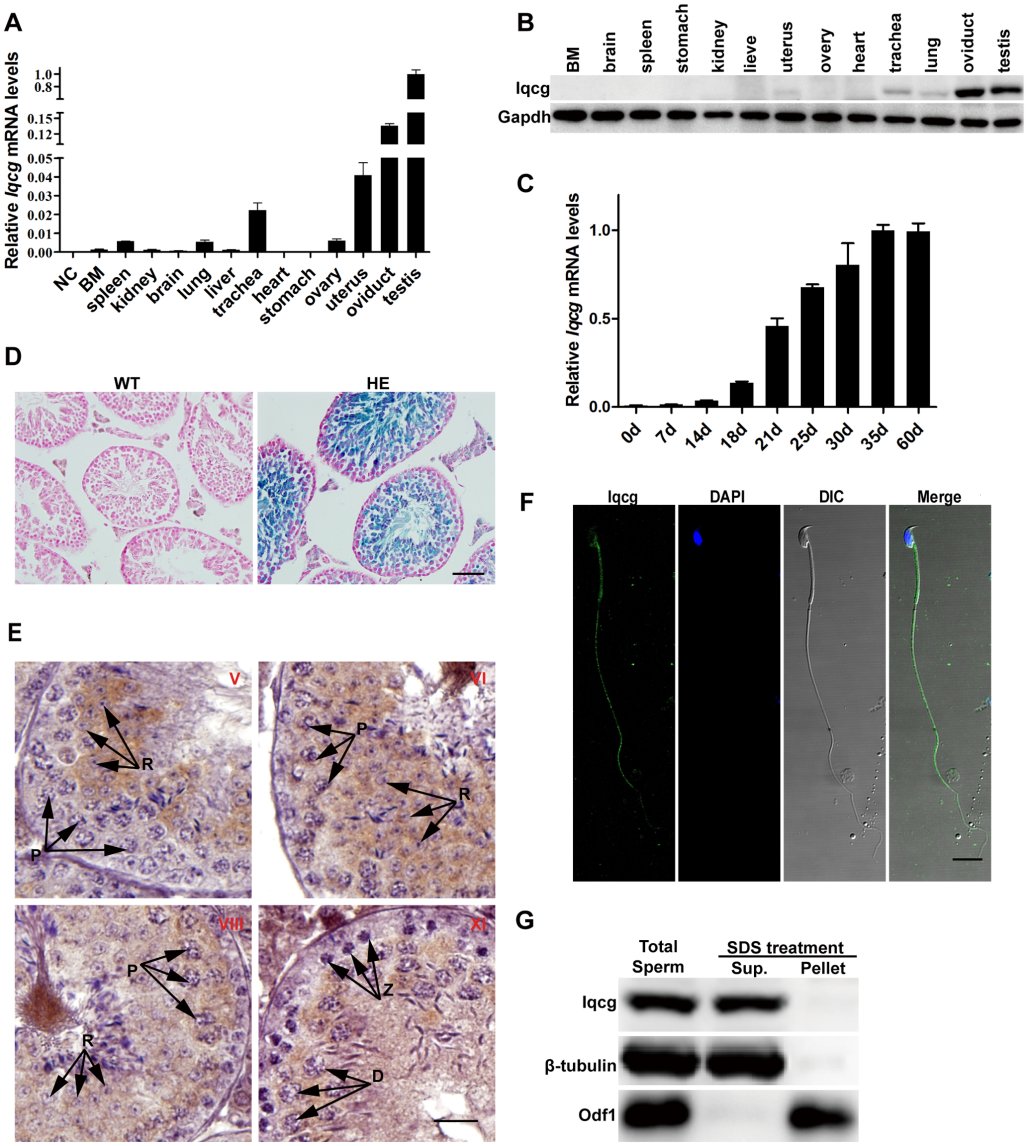
*Iqcg* was highly and orderly expressed in spermatogenesis of mice. (A) *Iqcg* mRNA levels in different tissues analyzed by quantitative RT-PCR using total RNA extracted from different tissues of mice. BM: bone marrow. Products of reverse transcription reaction with no mRNA templates were used as negative control (NC). Experiments were performed in triplicate and data were presented as means ± SEM. (B) Expression of Iqcg protein in different tissues. Gapdh was used as a loading control. (C) *Iqcg* mRNA levels in the first wave of spermatogenesis of mice. (D) X-gal staining of WT and *Iqcg* HE (heterozygote) testis sections. The X-gal reaction product (blue stain) was abundant in the spermatocytes and spermatids. Nucleus was counterstained with nuclear fast red. Scale bar  = 50 µm. (E) Immunohistochemical analysis of Iqcg protein in the WT testis sections. In stage V section, Iqcg was observed in the spermatids but not in spermatogonia or spermatocytes. Iqcg began to appear in the pachytene spermatocytes at stage VI. This signal persisted and was finally localized to the flagella of mature spermatozoa of testis, which can be seen in the lumens of testis sections at stage VI and stage VIII. Stage XI section showed Iqcg expression in diplotene spermatocytes but not in zygotene spermatocytes. The stages of seminiferous epithelial cycle were denoted by the Roman numerals. Different cell types of spermatocytes were indicated by the arrows. P: pachytene spermatocyte; Z: zygotene spermatocyte; D: diplotene spermatocyte; R: round spermatid. Scale bar  = 20 µm. (F) Immunofluorescence analysis of Iqcg on the slides of epididymal sperm. Iqcg was localized in the flagellum and post acrosomal region of sperm head. DAPI was used to stain nucleus. Scale bar  = 10 µm. (G) Western blot analysis of the sperm fractions after SDS-EDTA treatment. Iqcg was retained in the soluble fraction after this treatment. β-tubulin and Odf1 were used as controls for the soluble (Sup.) and SDS-resistant (Pellet) fractions respectively.

Quantitative reverse transcription-polymerase chain reaction (RT-PCR) assay was used to examine the expression levels of *Iqcg* in cDNA samples from 13 murine tissues. As previously reported in humans, *Iqcg* was ubiquitously expressed in various tissues (Fig. 1A). Interestingly, when compared with other tissues, the expression level of *Iqcg* was prominently enriched in the testis, which implied its potential role in spermatogenesis. To study the expression and localization pattern of Iqcg protein, a rabbit polyclonal antibody against the C-terminal fragment of murine Iqcg (amino acids 241–419) was generated and affinity purified. The specificity of this antibody was further affirmed using our KO mouse model (Fig. S1). Western blot analysis revealed that Iqcg protein was abundantly expressed in the testis and oviduct, followed by the trachea, lung, and uterus, consistent with the RT-PCR data (Fig. 1B).

Considering the high expression level of *Iqcg* in the testis, we paid more attention to the possible function of *Iqcg* in spermatogenesis. In mice, it is well known that the first wave of spermatogenesis takes place during 35 days post partum (dpp) [22], with particular types of germ cells emerging at a given time. Testes from male mice of different dpp were dissected and *Iqcg* mRNA levels were determined by quantitative RT-PCR method. As shown in Fig. 1C, *Iqcg* was first induced at 14 dpp, which was the time when pachytene spermatocytes appeared [22]. Then, the expression level of *Iqcg* went on to increase and reached the peak at 35 dpp, at which time the first wave of spermatogenesis completed. The expression of *Iqcg* in spermatocytes and spermatids were further verified by X-gal staining assay later using our KO mouse model (Fig. 1D). Moreover, immunohistochemical analysis of sections of adult mouse testis showed Iqcg protein was first detected in the cytoplasm of pachytene spermatocytes of stage VI. The signal persisted and was finally localized to the flagellum of mature testicular spermatozoon (Fig. 1E). Indirect immunofluorescence assay of epididymal spermatozoa also showed Iqcg protein localization in the flagellum and post-acrosomal region of the head (Fig. 1F). To further determine the biochemical nature of Iqcg in spermatozoa, we treated epididymal spermatozoa with SDS-EDTA solution, which is known to be unable to dissolve the accessory structures of the sperm flagellum, such as mitochondrial sheath, outer dense fibers and fibrous sheath [23]. Iqcg was retained in soluble supernatant after this treatment, suggesting Iqcg was not a component of these accessory structures (Fig. 1G).

**Figure 2:**
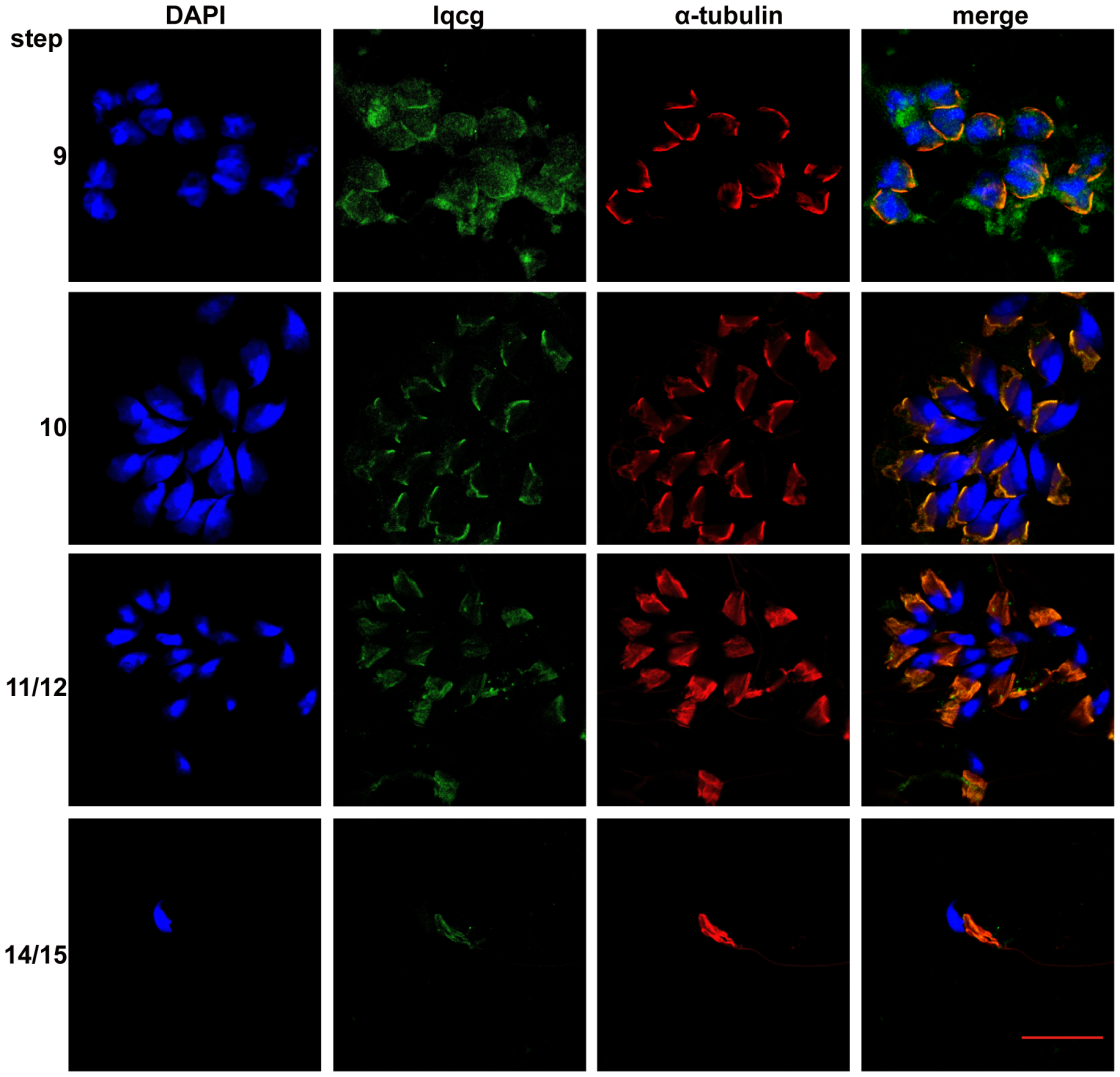
Iqcg was associated with manchette in the developing spermatids. Immunofluorescence was performed on the drying down preparations corresponding with spermatids of different steps. α-tubulin was used as a marker for manchette. DAPI was used to stain nucleus. Scale bar  = 20 µm.

To further study the possible role of Iqcg in spermiogenesis, the subcellular localization of Iqcg in the developing spermatids on stage-specific drying down preparations was investigated. Interestingly, Iqcg displayed a colocalization with manchette (Fig. 2), a testis-unique and transient microtubular structure which is believed to play a central role in the nuclear shaping as well as flagellum formation by the intramanchette transport (IMT) process [24], [25], [26]. These results further suggested that Iqcg might be a structural component of sperm flagellum that was transported along manchette, or play a role in IMT by either affecting the structural integrity of manchette or regulating the transport process.

### Generation of *Iqcg* knockout mice

**Figure 3:**
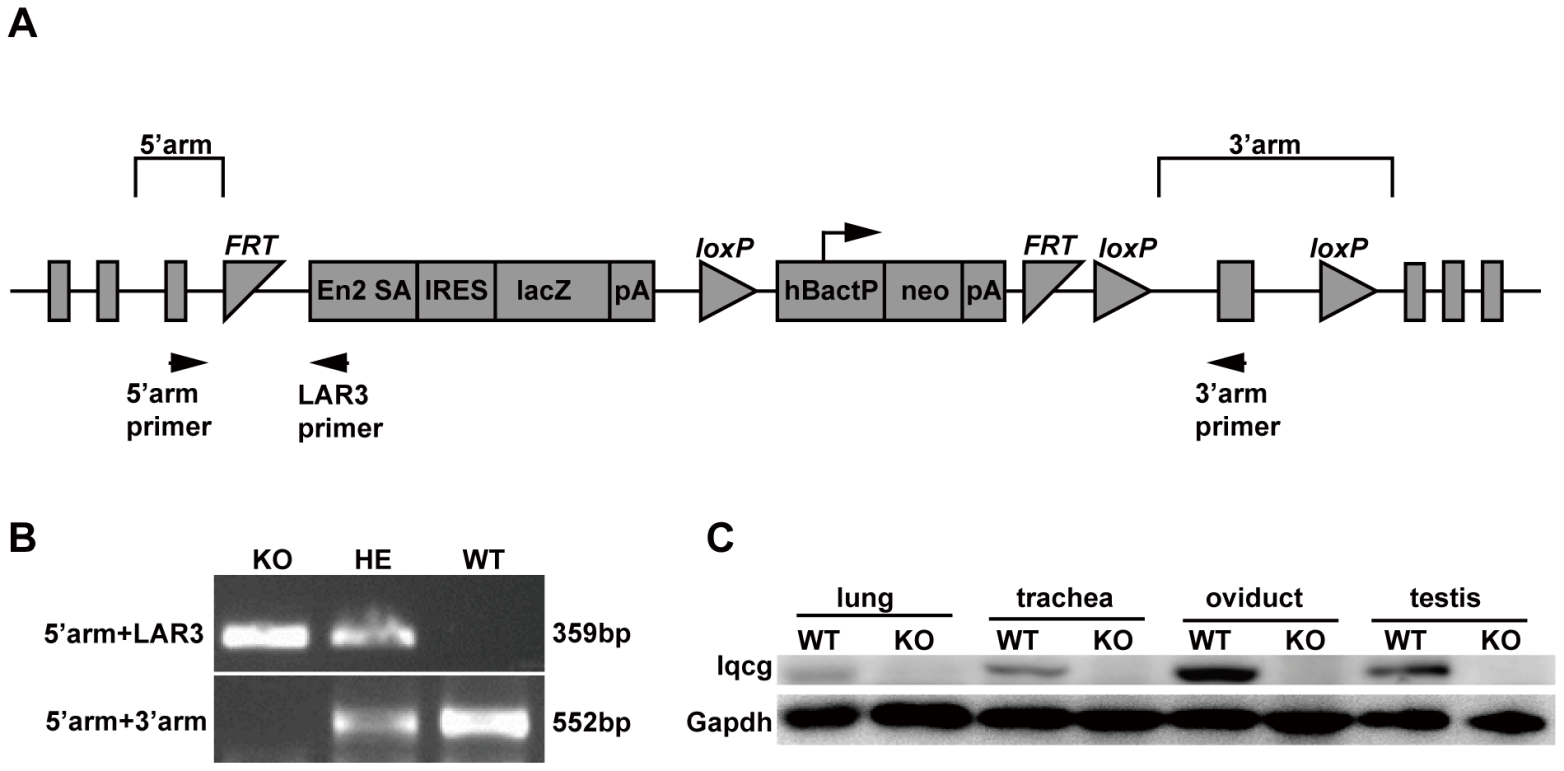
Generation of *Iqcg* knockout mice. (A) Schematic of the “knockout first” targeting strategy used for generating *Iqcg* KO mice. This strategy introduced an Flp-recombinase target (FRT)-flanked selection cassette into the intron after the fifth exon of *Iqcg*, which could trap the transcript through the Engrailed-2 splice acceptor (En2 sA) element and truncate it through the SV40 polyadenylation signal (pA). LacZ encoding β-galactosidase was a reporter gene that could be used to trace the expression pattern of the *Iqcg*. Neomycin resistance gene (Neo) was used as a marker for ES clone screening which was driven by an autonomous promoter (hBactP). IRES: internal ribosome entry site. (B) A representative genotyping by PCR assay using primers specific for mouse *Iqcg* (5′arm and 3′arm) and LAR3 (designed on the En2 SA element of the targeted allele). HE: heterozygote. (C) KO efficiency examined by Western blot analysis in representative tissues.

The high and ordered expression pattern of *Iqcg* in mouse testis strongly indicated that it might be involved in spermatogenesis. To address this hypothesis, and also to explore other possible physiological functions of *Iqcg* in mice, we generated *Iqcg* KO mouse model using targeted ES cell clone from European Conditional Mouse Mutagenesis Program (EUCOMM). This KO mouse model was based on the “knockout first strategy” of gene targeting (Fig. 3A) [27]. The strategy introduced an Flp-recombinase target (FRT)-flanked selection cassette into the intron after the fifth exon of *Iqcg*, which could trap the transcript through the Engrailed-2 splice acceptor (En2 sA) element and truncate it through the SV40 polyadenylation signal (pA). LacZ encoding β-galactosidase was a reporter gene that could be used to trace the expression pattern of the *Iqcg* (Fig. 3A and 1D). Genotyping was performed as indicated to distinguish the WT, heterozygous, and homozygous KO mice (Fig. 3A and 3B). The KO efficiency of this targeting strategy was confirmed in the representative tissues using Western blot analysis (Fig. 3C). As this KO strategy was based on the pre-termination of transcription, there might exist a truncated form of Iqcg in the KO mice. We designed primers on the 5′region of *Iqcg* and confirmed that such truncated form of *Iqcg* mRNA indeed existed (Fig. S2). However, the potential truncated protein contained no structural domains and should be considered as “loss of function”. The KO mice were viable and showed no gross abnormalities when compared with WT controls. The histology of major organs of KO mice, except for testis and epididymis, was normal (Fig. S3). The ratio between three genotypes accorded with Mendel pattern of inheritance, suggesting *Iqcg* was not essential for embryonic development.

### *Iqcg* knockout caused male infertility

Mating experiment was performed to evaluate the consequence of *Iqcg* KO on male fertility. Adult WT and KO male mice (10–12 weeks) were continuously coupled with WT female mice in the ratio of 1∶2 during 4 weeks, with the females changed once a week. Both the WT and KO males could plug the females in a comparable frequency, indicating the mating behavior of the KO mice remained unaffected (Table 1). However, none of the females mated with KO males became pregnant and gave birth to any offspring (Table 1), demonstrating the complete infertility of the KO male mice. However, female fertility appeared unaffected in the KO mice, as they could become pregnant and give birth to a normal litter size like the WT controls (Table 1).

**Table 1:** *Iqcg* knockout caused male infertility.

	Males	Females	Plugs	Pregnancies	Average litter size
**Male fertility test**	WT(*n* = 6)	WT(*n* = 48)	38	36	7.5
	KO(*n* = 6)	WT(*n* = 48)	33	0	0
**Female fertility test**	WT(*n* = 5)	WT(*n* = 10)	8	7	5.6
	WT(*n* = 5)	KO(*n* = 10)	9	7	5.5

### *Iqcg* was required for sperm flagellum formation

**Figure 4:**
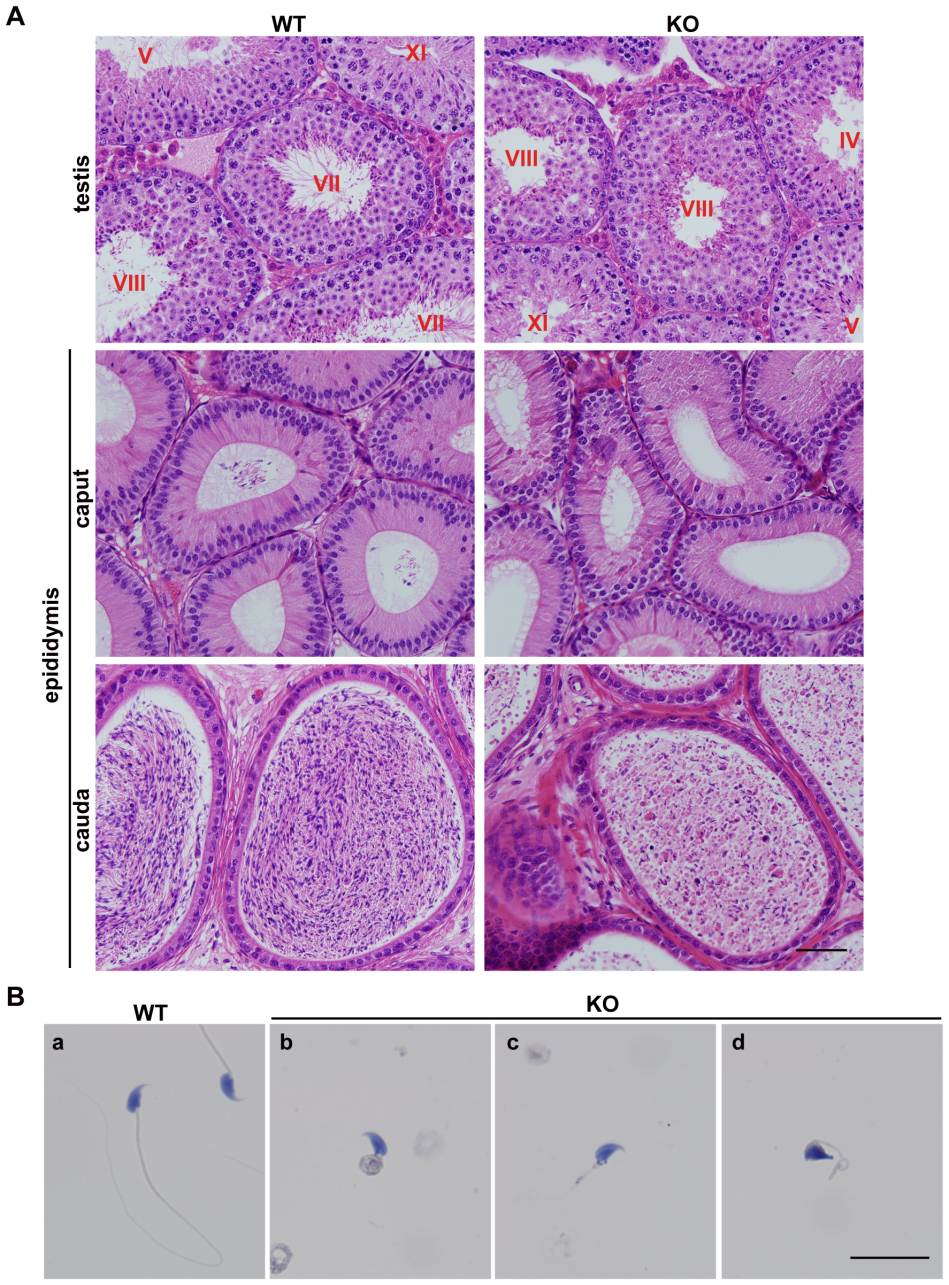
*Iqcg* was required for sperm flagellum formation in mice. (A) H&E staining of WT and *Iqcg* KO testis and epididymis sections. In the WT testis, the lumens of seminiferous tubules of stage VII-VIII were filled with flagella. However, in the KO mice, no visible flagella could be found. The caput of WT epididymis displayed some spermatozoa in the lumens, but no spermatozoa were observed in the KO caput epididymis. WT cauda epididymis were filled with normal spermatozoa with long tails. However, the KO cauda epididymis only contained malformed spermatozoa and degenerated cell debris. The stages of seminiferous epithelial cycle were denoted by the Roman numerals. Scale bar  = 50 µm. (B) H&E staining of WT and *Iqcg* KO spermatozoa on slides. WT spermatozoa showed a thin and long flagellum (a). Most KO spermatozoa showed an extremely short tail and were often connected by a mass of cytoplasm (b). Some KO spermatozoa only showed a short tail (c). A part of KO spermatozoa also displayed nuclear shaping defects (d). Scale bar  = 20 µm.

Detailed analyses were conducted to explore the reasons accounting for the male infertility of the *Iqcg* KO mice. First, we compared the testis weight, body weight, and testis/body weight ratio between WT and KO mice. No significant differences of these parameters were found (Table S1). Histological analysis of adult testis sections revealed that the spermatogonia, spermatocytes, and round spermatids of the KO mice remained normal both in quantity and morphology (Fig. 4A), suggesting that the mitotic and meiotic phases of spermatogenesis were not affected by *Iqcg* KO. This presumption was also supported by the histological analysis of the testis of 7dpp (only with spermatogonia), 14dpp (with spermatogonia and spermatocytes), and 21dpp (with spermatogonia, spermatocytes and round spermatids), which showed no significant differences between WT and KO mice (Fig. S4). However, while the lumens of adult WT seminiferous tubules of stage VII-VIII were filled with flagella, none of the KO seminiferous tubules contained any visible flagella (Fig. 4A). The lumens of the cauda epididymis were filled with mature spermatozoa with long tails in the WT mice, whereas in the KO mice, these normal sperm tails could never be found (Fig. 4A). In addition, a large amount of degenerated cell debris was observed in the KO cauda epididymis (Fig. 4A).

The morphological defects of the KO spermatozoa can be observed more distinctly on the slides. All KO spermatozoa were identified by their extremely short flagella and often associated with a thick irregular cytoplasm (Fig. 4B). Nuclear shaping defects were also observed in 18.3%±2.9% of KO spermatozoa (versus 3.0%±0.6% in WT spermatozoa, mean ± SEM, *n* = 3 for mice of each genotype), in which the nuclei displayed irregular shapes and lost the typical hook-shape appearance (Fig. 4B and Fig. S5). While the WT spermatozoa showed motilities of different degrees, the KO spermatozoa displayed no motility at all, consistent with their paralyzed tails (Movies S1 and S2). Collectively, these results demonstrated that *Iqcg* was required for the normal sperm flagellum formation and *Iqcg* KO caused severe malformation and total immobility of the spermatozoa.

To further identify the defects of spermiogenesis, we used acetylated α-tubulin as a marker for sperm flagellum axoneme to trace the flagellum formation in the KO spermatids. As shown in Fig. S6, flagellum formation was already disrupted in the elongating spermatids, which showed very short axonemes and these axonemes were sometimes abnormally frizzled.

It is known that cytoskeletons, especially for microtubules and actin filaments, are critical for spermiogenesis [24], [28], [29]. Microtubules are major components of manchette, which plays essential roles both in head shaping and cargo transport to the tail region in developing spermatids [24], [25]. In several mouse models of spermiogenesis defects, manchette was found to disappear or to be abnormally assembled [17], [19], [30], [31]. Actin filaments are enriched in the acrosome-acroplaxome region and their dynamics plays important roles in head shaping [28], [32]. Furthermore, actin filaments, as well as motor proteins based on them, were also reported to exist in the manchette structure and supposed to assist the microtubular system in cargo transport [32]. We asked whether the spermiogenesis defects in *Iqcg* KO spermatids might arise from the failure of assembly of these structures. We used antibodies against α-tubulin and β-actin to stain these structures. As seen from Fig. S7, actin filaments were prominently found in the acrosome-acroplaxome region, while α-tubulin defined the manchette structure. Actin filaments were also found in the manchette, although the signal was weaker than that in the acrosome-acroplaxome region, consistent with the previous report [32]. However, when compared with the WT controls, both the microtubules and actin filaments were kept intact after *Iqcg* KO. These results suggested that Iqcg did not participate in spermiogenesis through regulating the assembly of these cytoskeletal structures.

### Ultrastructural characterization of *Iqcg* knockout spermiogenesis

**Figure 5:**
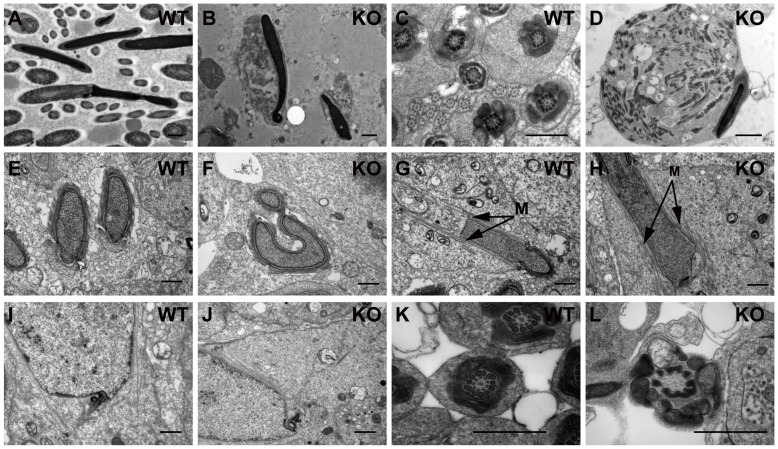
Ultrastructural analysis of *Iqcg* knockout spermiogenesis. (A) WT epididymis sections showed different segments of sperm flagellum. (B) In *Iqcg* KO epididymis, no normal flagellum structures were found. The sperm head was often associated with a mass of cytoplasm where components of flagellum were found but could not be assembled correctly. (C) WT section of testis at stage VIII, which displayed the well-organized structures of middle piece, principle piece and end piece of step 16 sperm flagellum. (D) *Iqcg* KO step16 spermatid, showing similar malformation as the epididymal sperm. (E) A cross section of two heads of step 11 spermatids showing the normal nuclear shape. (F) A step 12 KO spermatid showed malformed head shape. The cross section in the acrosome region of this spermatid displayed distorted shape of the nucleus and the acroplaxome. (G) WT step 11–12 spermatid, showing the well assembled manchette (indicated by arrows and “M”). (H) KO spermatid at step 11–12, showing normal manchette structure. (I) WT step 9 spermatid showed that the centriole had been located to the posterior region of the nucleus. (J) Centriole in KO step 9 spermatid also showed successful migration to posterior region of the nucleus. (K) Cross section of WT step 16 spermatid at middle piece, showing the regular arrangement of mitochondria, outer dense fibers and microtubules. (L) Cross section of KO step 16 spermatid at middle piece, showing mitochondria and outer dense fibers, but the inner “9+2” arrangement of microtubules were missing. Scale bar  = 1 µm.

To characterize the abnormalities of *Iqcg* KO spermiogenesis at the ultrastructural level, transmission electronic microscopy (TEM) analyses of testis and epididymis from adult WT and KO mice were performed. In the WT epididymal spermatozoa, normal “9+2” pattern of the axonemal microtubules, as well as other accessory elements such as outer dense fibers, mitochondrial sheath and fibrous sheath were found in a well-organized arrangement (Fig. 5A). However, none of these elegant configurations were found in the KO epididymis (Fig. 5B). Instead, the sperm heads were often associated with an irregular mass of cytoplasm, in which the components of flagellar structures were scattered and could not be assembled correctly (Fig. 5B).

For the step 16 spermatids, representing the most mature spermatids in testis and ready to be released to the lumen of seminiferous tubule, the morphological defects were quite similar with the epididymal spermatozoa, showing the mass of cytoplasm with disorganized structures of normal flagella (Fig. 5C and 5D). Consistent with the results of sperm slides, nuclear shaping defects were also observed in some developing KO spermatids (Fig. 5E and 5F). Moreover, normal manchette structures were found in most elongating *Iqcg* KO spermatids (Fig. 5G and 5H), in agreement with the α-tubulin staining result.

Attention was also paid to the centrioles of developing spermatids, as they would form the basal body of the normal axoneme. Both in the WT and KO spermatids, centrioles were found and located to the posterior region of the nucleus around step 9 (Fig. 5I and 5J). Despite the normal location of the centrioles in the KO spermatids, the extending of the axoneme from these centrioles appeared to be disturbed. As a result, the normal “9+2” pattern of the axoneme could be hardly found. In some cross sections of the extremely short tails, the mitochondrial sheath appeared to surround the outer dense fibers, but with no microtubular structures in the inner side, reflecting the failure of axoneme growth (Fig. 5K and 5L).

### Iqcg interacted with calmodulin in a calcium ion concentration dependent manner in the testis

**Figure 6:**
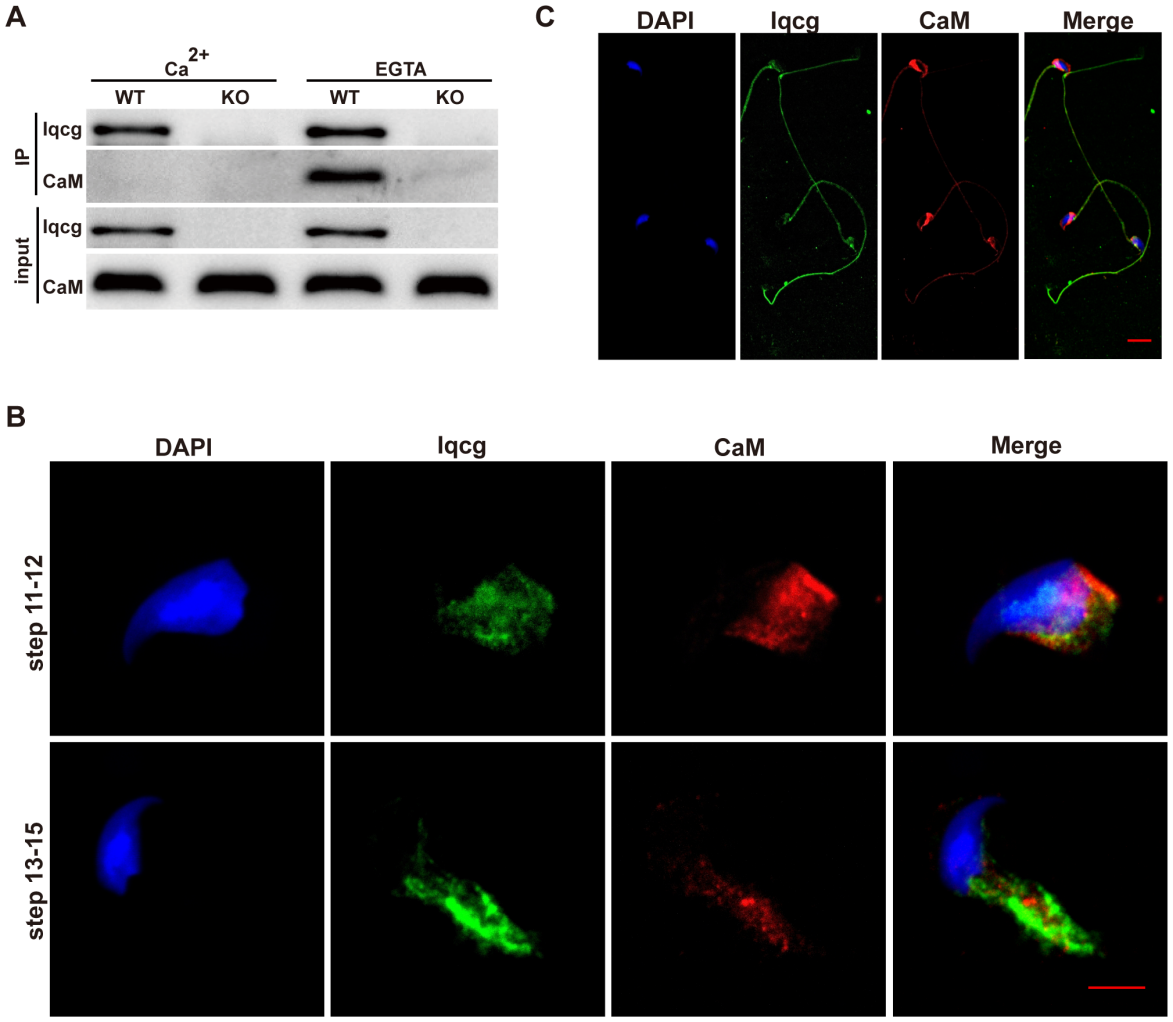
Iqcg interacted with calmodulin in testis in a calcium concentration regulated manner. (A) Co-immunoprecipitation was performed in the cell lysates from WT and *Iqcg* KO testis using Iqcg antibody. Western blot analyses were conducted with Iqcg and calmodulin antibody. (B) Both Iqcg (green) and calmodulin (red) existed in the manchette region of elongating spermatids. DAPI was used to stain nucleus. Scale bar  = 5 µm. (C) Iqcg (green) and calmodulin (red) localization in epididymal spermatozoa. Both Iqcg and calmodulin were found in the flagellum and post-acrosomal region. DAPI was used to stain nucleus. Scale bar  = 10 µm.

It is known that some IQ motif containing proteins interact with calmodulin in a calcium ion concentration dependent manner [33]. Here, we addressed whether Iqcg also interacted with calmodulin endogenously in the context of spermatogenesis. As shown in Fig. 6A, calmodulin was co-immunoprecipitated with Iqcg in the mouse testis when the calcium ion was chelated by 2 mM EGTA. While in the presence of 2 mM CaCl_2_, this interaction was abrogated. Immunofluorescence analysis also showed in the elongating spermatids, Iqcg and calmodulin displayed some colocalization, both of which were found in the manchette region (Fig. 6B). Furthermore, in mature epididymal spermatozoa, both Iqcg and calmodulin were found in the flagellum and post-acrosomal region, although calmodulin was more enriched in the post-acrosomal region than Iqcg (Fig. 6C). The co-existence of Iqcg and calmodulin in these regions brought the possibility for them to physically interact with each other in a particular cell context with a certain intracellular calcium level. These results suggested that Iqcg may function through calmodulin and its downstream molecules to participate in spermiogenesis and the interaction between Iqcg and calmodulin may also exist in mature spermatozoa.

### Other cilia structures were not affected by *Iqcg* knockout

Apart from sperm flagellum, axoneme structure also exists in cilium, a subcellular structure present in nearly all cell types in vertebrate. Cilia play important roles either in sensing extracellular signals or propelling fluid flow. Defects of ciliogenesis can cause a lot of symptoms such as obesity, polydactyly, retinal degeneration, hydrocephalus and left-right asymmetry defect [34], [35], [36]. Since Iqcg was also highly expressed in tissues with motile cilia, e.g., trachea and oviduct, we asked whether Iqcg played a role in ciliogenesis in these tissues. Interestingly, immunohistochemical analysis showed Iqcg was also localized in the cilia of trachea and oviduct (Fig. S8), implying the potential role of Iqcg in the formation of these cilia. However, in contrast with the collapse of sperm flagella, the structures of cilia from the trachea and oviduct of *Iqcg* KO mice appeared normal, both in histological and TEM analyses. The lengths of these cilia were comparable to the WT control, and the “9+2” pattern of microtubular arrangement was kept intact (Fig. S9). Consistent with their normal structures, rapid movements of these cilia from KO trachea and oviduct *in vitro* were observed (Movies S3-S6). Furthermore, symptoms like obesity, polycystic kidney, hydrocephalus, or left-right asymmetry defect were not found in the KO mice, suggesting that Iqcg was not indispensable for the formation of other cilia.

## Discussion

Sperm flagellum is a highly specialized structure that is essential for sperm motility. Although the molecular mechanisms underlying flagellum formation are poorly understood, many studies from KO mouse models have begun to uncover genes essential for this process [5], [37]. Among them some genes are also responsible for other ciliary defects, as shown in the cases of *Bbs2*-, *Mkks*-, *Mns-1*- and *Kif3a*-deficient mouse models [14], [19], [38], [39]. In this report, we demonstrated that *Iqcg*, which first attracted our attention for its involvement of chromosome translocation of human leukemia, was critical for spermiogenesis in mice, especially for the flagellum formation. The phenotype of *Iqcg* KO mice appeared testis-specific, as other cilia structures, as well as the histological appearances of other major tissues, remained unaffected. Our results here were generally consistent with the previous report of *Iqcg*-mutant mice [21], although there are some differences of the phenotypes when compared in detail. First in our model, all spermatozoa displayed malformation, especially the failure of normal flagellum formation, while in the previously reported *Iqcg*-mutant mice, a small part (about 3%) of spermatozoa remained normal appearance. Second, the defect of sperm flagellum formation was seemingly more severe in our KO model, as a part of spermatozoa in *Iqcg*-mutant mice can develop long flagella, although these flagella were often detached from the sperm heads. We speculate such differences are possibly caused by the remaining low level of WT transcript in that mutant mouse model, which may contribute to form the apparently normal flagella but with deficient package of internal flagellum structures and thus will be easy to be detached from the sperm heads. However, they further generated *Iqcg* KO mice, also using an ES cell clone from EUCOMM. The only difference of those KO mice to ours is that they additionally deleted the sixth exon by crossing with Cre transgenic mice, probably to avoid the potential failure of transcription termination by pA element provided in the targeting cassette. However, in our study, we found the targeting cassette was effective to truncate the transcription of *Iqcg*. Thus, from this point of view, these two KO models would have identical phenotypes, although in that report, detailed comparison between their KO and mutant mice were not carried out.

Although the spermiogenesis defects of spermatids in this setting were carefully checked, the exact mechanism of Iqcg in regulating of spermiogenesis is still unclear. It is believed that manchette, a transient microtubular structure emerging in the elongating phase of spermiogenesis, is critical for sperm head shaping, as well as flagellum formation [24]. IMT model was proposed, by which protein complex and organelles could be transported to the posterior part of spermatids by motor proteins along the manchette microtubules [25]. Several genes essential for spermiogenesis are also important for the regulation of manchette assembly, such as *Ift88*, *RIM-BP3*, *Meig-1*, and *Kif3a* [17], [19], [30], [31]. Moreover, another cytoskeleton, actin filaments were also reported to be important in sperm head shaping and may also assist the microtubular system to transport cargos in the manchette [32]. In our *Iqcg* KO mouse model, most elongating spermatids showed normal structures of both microtubules and actin filaments, excluding that *Iqcg* is required for the assembly of these cytoskeletal elements. Considering the colocalization of Iqcg and manchette in developing spermatids, there are two reasonable speculations of Iqcg's molecular function. First, Iqcg may be a structural component of sperm flagellum transported by IMT process, whose deficiency can cause the failure of assembly of the axoneme. Second, Iqcg may play some roles in regulating IMT process, as the defects of cargo transport may also cause spermiogenesis defects. Interestingly, Iqcg was also highly expressed in tissues with motile cilia, e.g., trachea and oviduct. In these tissues, Iqcg was also localized in the cilia, consistent with previous categorization of Iqcg to ciliary proteins, using bioinformatical approaches [40]. However, these cilia structures were kept normal after *Iqcg* KO, suggesting that Iqcg's role in these cilia might be compensated by other related molecules. On the other hand, this observation raised the possibility of Iqcg in regulating IMT process, as IMT is an unique process required for sperm flagellum formation, but not for ciliogenesis in other tissues.

Calcium signaling was well studied in the regulation of sperm motility and acrosome reaction, whereas its role in the spermiogenesis remains enigmatic [41]. Previous studies mainly focused on the biochemical and electrophysiological properties of the calcium channels existing in the spermatogenic cells and the expression and localization pattern of the molecules related to calcium signaling, such as calmodulin [42]. Calmodulin is also highly expressed in the testis and displays a regulated expression pattern during spermatogenesis [43], [44], [45]. In the spermatids, calmodulin was reported to be localized to many sites including the acrosome, flagellum and post acrosomal sheath [46], [47], [48]. It was also found in the perinuclear ring region of the developing spermatids, from where the manchette microtubules develop [48]. The regulating roles of calmodulin in cytoskeletal dynamics, both for microtubules and actin filaments, were well established [49], [50], [51]. Furthermore, calmodulin also plays important roles in regulating the activity of unconventional myosins, e.g., myosin Va, which has also been implicated in the IMT process along actin filaments [32], [52]. However, so far there is still no direct evidence of calcium signaling in regulating spermiogenesis. Our study here suggested that Iqcg might participate in spermiogenesis through its dynamic interaction with calmodulin in different intracellular calcium levels. Although the functional significance of this dynamic interaction is still unclear, this result further indicated that calmodulin and its downstream pathways might be important for spermiogenesis.

Interestingly, in clinic, the short tail or stump tail defects of spermatozoa in human male infertility have been reported, whereas the genetic causes of these defects have not yet been identified [53], [54]. These patients displayed poor sperm flagellum formation and the ultrastructures of the sperm tails were very similar to the sperm phenotypes observed in our *Iqcg* KO mice, which suggested *Iqcg* might be a good candidate gene for these short/stump sperm tail defects in human.

In summary, our report here indicates that *Iqcg* is essential for the sperm flagellum formation in mice. Iqcg is associated with manchette in the developing spermatids and might play its role through calcium signaling. These results will not only help us to further understand the molecular mechanisms underlying the sperm flagellum formation but also contribute to uncover the genetic origins of the short/stump tail defects in human male infertility.

## Materials and Methods

### Ethics statement

All protocols for animal maintaining and handling were approved by The Laboratory Animal Ethics Committee of Rui-Jin Hospital affiliated to Shanghai Jiao Tong University School of Medicine (Permit Number: 048). All efforts were made to minimize suffering of animals.

### mRNA expression analyses

Tissues were collected, briefly washed with PBS, and then immediately homogenized in TRIZOL Reagent (invitrogen) on ice. RNA extraction was carried out as standard protocol. 1 µg total RNA sample was reverse transcribed using moloney murine leukemia virus (M-MLV) Reverse Transcriptase (Invitrogen). cDNA was amplified using primers for *Iqcg*: 5′-TGGAGGAGATTGAGAAACTGAG-3′ (forward) and 5′- GCCAGGTCTTGCAGGTGTAC-3′ (reverse). *Gapdh* was co-amplified as an endogenous control, with primers 5′-CCTGGAGAAACCTGCCAAGTAT-3′ (forward) and 5′- GGTCCTCAGTGTAGCCCAAGAT-3′ (reverse). For detecting the potential truncated mRNA in the KO mice, two independent pairs of primers, named *Iqcg* 5′-1 (forward: 5′-GAGGTGGAAGTCGTGGAGT-3′; and reverse: 5′-GGAATGATGTAGCCGAGAATA-3′) and *Iqcg* 5′-2 (forward: 5′-GGGCAGGACTTTACCTTTA-3′; and reverse: 5′-GAGCCAGAATCTTGCATCTC-3′) were designed in the 5′ region of *Iqcg*. For semi-quantitative RT-PCR, cDNA was amplified using Recombinant *Taq* DNA Polymerase (Takara) following the manufacturer's instructions. For quantitative RT-PCR, cDNA was amplified using a SYBR Green PCR Master Mix (Applied Biosystems) with Applied Biosystems 7500 Real-Time PCR System in triplicate for each sample. The relative expression levels to *Gapdh* were calculated using cycle time (Ct) values and the equation: relative quantity = 2^−ΔCt^. The obtained results were then normalized to corresponding percentages of highest expression level.

### Western blot

Tissues were homogenized in PBS and then lysed with 5× SDS-PAGE sample buffer by sonication for 5 minutes and boiling for 10 minutes. Samples were separated by SDS-PAGE and transferred to PVDF membranes (GE Healthcare). Membranes were blocked with 5% skim milk for 1 hour, followed by incubation with the primary antibodies at 4°C overnight. Primary antibodies used were as follows: rabbit polyclonal antibody against Iqcg (1∶500 dilution, generated by this study), rabbit monoclonal antibody to GAPDH (1∶1000 dilution, code #2118, Cell Signaling Technology), mouse monoclonal antibody to Calmodulin (1∶500 dilution, code 05-173, Millipore). After washed with TBST buffer (20 mM Tris-HCl, pH 7.6, 150 mM NaCl, and 0.1% Tween-20) for three times, membranes were incubated with HRP-conjugated secondary antibodies (Cell Signaling Technology) for 1 hour. After washed with TBST for three times, specific proteins bands were visualized using Immobilon Western Chemiluminescent HRP Substrate (Millipore) with a Fujifilm LAS-4000 system.

### Generation of *Iqcg* knockout mice

*Iqcg*-targeted C57BL/6N ES cell clone was ordered from the European Conditional Mouse Mutagenesis Program (Project ID: 36432; ES cell clone number: EPD0105-3-A02; Further quality control data and protocols for generating this targeted ES cell clone can be found at http://www.mousephenotype.org/martsearch_ikmc_project/martsearch/ikmc_project/36432 and http://www.i-dcc.org/kb/entry/82/, respectively) and injected into C57BL/6 blastocysts. The resulting male chimeras were mated with C57BL/6 females to get *Iqcg* heterozygotes. Heterozygotes were inbred to obtain *Iqcg* homozygotes. Genotyping was performed by PCR with primers specific for mouse *Iqcg* (5′arm: 5′-CGCCTTCCACCGCGTAGTTCTGGG-3′ and 3′arm: 5′-CAGGTTCTAGGGCTAGAGAATGGC-3′) and LAR3 (5′-CACAACGGGTTCTTCTGTTAGTCC-3′) to amplify a 552-bp fragment from the WT allele or a 359-bp fragment from the targeted allele. The PCR was performed at 94°C for 3 minutes and followed by 35 cycles at 94°C for 30 seconds, 58°C for 30 seconds, and 72°C for 45 seconds, and 1 cycle at 72°C for 10 minutes.

### Generation and purification of Iqcg antibody

Mouse Iqcg protein fragment (amino acids 241–419) was expressed in Escherichia coli and purified to immunize rabbits to get antiserum. Antigen affinity purification was performed using CNBr-activated Aogarose 4B (AOGMA) as standard protocol.

### Histology

For histology, testis, epididymis, trachea and oviduct were fixed in Bouin's solution overnight at 4°C. For brain, mice were perfused with 4% paraformaldehyde from the heart. Other tissues were fixed in 4% formaldehyde. Samples were processed, sectioned, and stained with hematoxylin and eosin.

### Drying down preparations

Drying down preparation was performed generally as previously reported [19]. Mouse testes were dissected and decapsulated in PBS. Specific stages of the seminiferous tubules were identified by transillumination [55] and stage-specific segments were transferred in 100 mM sucrose solution. Stage specific cell suspension was spread on an object slide and was air dried at room temperature and stored at −80°C.

### Immunohistochemistry and immunofluorescence

Bouin's solution-fixed, paraffin-embedded tissue sections were rehydrated, antigens were retrieved by pressure cooking in 10 mM sodium citrate (pH 6.0) for 8 minutes, and nonspecific binding sites were blocked in 1% BSA. Endogenous peroxidase activity was blocked in 3% H_2_O_2_. Sections were then incubated with Iqcg antibody diluted 1∶1000 in blocking solution overnight at 4°C. After washed with PBS, section was incubated with ChemMate DAKO EnVision/HRP for 1 hour at room temperature, washed with PBS, and detected by the ChemMate DAB+ Chromogen. The section was counterstained with hematoxylin, dehydrated and mounted in Neutral balsam.

Immunofluorescence on sperm slides was performed as previously reported [23]. Epididymal spermatozoa were collected, attached to slides, and fixed with 4% paraformaldehyde for 15 minutes. After washed with PBS, spermatozoa were permeabilized with −20°C methanol for 2 minutes. The slides were washed with PBS, and the samples were incubated with 1% BSA for 30 minutes at room temperature and then with antibodies (anti-Iqcg, 1∶50 dilution; mouse anti-calmodulin, 1∶50 dilution, code ab2860, Abcam) diluted in blocking solution for 4°C overnight. The samples were incubated with FITC-conjugated Goat Anti-Rabbit IgG (H+L) (Jackson ImmunoResearch) diluted 1∶500 in blocking solution for 1 hour at room temperature. After washed with PBS, the samples were mounted with cover slips using VECTASHIELD Mounting Medium with DAPI (VECTOR).

For immunofluorescence on drying down preparation, slides were fixed in 4% PFA for 15 minutes and washed with PBS. Autofluorescence was quenched with 100 mM ammonium chloride for 2 minutes, and slides were subsequently washed with PBS and treated with 0.5% Triton X-100 for 5 minutes. After PBS washes slides were blocked with 10% normal goat serum in PBS for 2 hours. Primary antibodies (anti-Iqcg, 1∶500 dilution; mouse anti-α-tubulin, 1∶500 dilution, code T9026, Sigma; rabbit anti-α-tubulin, 1∶500 dilution, code #2125S, Cell Signaling Technology; rabbit anti-Acetylated-α-tubulin, 1∶500 dilution, code #5335, Cell Signaling Technology; mouse anti-β-actin, 1∶500 dilution, code A1978, Sigma; mouse anti-calmodulin, 1∶100 dilution, code ab2860, Abcam) were diluted in 3% normal goat serum and incubated at 4°C overnight. Slides were washed with 0.1% PBS-Tween 20 and incubated with FITC-conjugated Goat Anti-Rabbit IgG (H+L) or Rhodamine Red-X-conjugated Goat Anti-Mouse IgG (H+L) (Jackson ImmunoResearch) diluted 1∶500 in 3% goat serum for 1 hour at room temperature. After washed with 0.1% PBS-Tween 20, the samples were mounted with cover slips using VECTASHIELD Mounting Medium with DAPI (VECTOR).

### SDS-EDTA treatment of spermatozoa

SDS-EDTA treatment was performed as previously reported [39]. Epididymal spermatozoa were collected into PBS and centrifuged at 800 *g* for 5 minutes at RT. Spermatozoa were homogenized in 1 ml of SDS-EDTA solution (1% SDS, 75 mM NaCl, 24 mM EDTA, pH 6.0) and centrifuged at 5000 *g* for 30 minutes at RT. The supernatant and the pellet sample were respectively prepared in the same final volume with SDS-PAGE sample buffer for Western blot analysis. β-tubulin (1∶1000 dilution; code sc-55529, Santa Cruz) and Outer dense fiber-1 (Odf1; 1∶1000 dilution; code sc-27907, Santa Cruz) were used as controls for the soluble and SDS-resistant fractions respectively.

### Transmission electron microscopy

TEM was performed at the Laboratory of Electron Microscopy, Shanghai Jiao Tong University School of Medicine, following standard protocols. Mice were perfused with 2% glutaraldehyde from heart. Testis, epididymis, trachea and oviduct were dissected, and post- fixed in 1% osmium tetroxide. Dehydration was carried out in ethanol and the samples were embedded in Epon 812. Ultrathin sections were counterstained with uranyl acetate and lead nitrate, and examined with a PHILIPS CM120 transmission electron microscope.

### X-gal staining

Testes were dissected, fixed for 2 hours in 4% paraformaldehyde at 4°C, washed with PBS, and then incubated for 10–16 hours with X-gal solution. After being washed with PBS, testes were further fixed in 10% neutral buffered formalin overnight. They were then dehydrated, processed, and embedded in paraffin. Sections were cut and counterstained with nuclear fast red.

### Co-immunoprecipitation

Testis was decapsulated and digested using collagenase and trypsin. After washed with TBS (50 mM Tris-HCl (pH 7.4) and 150 mM NaCl), cells were lysed on ice for 30 minutes with lysis buffer (50 mM Tris-HCl (pH 7.4), 2 mM EGTA or CaCl_2_, 150 mM NaCl, 1% (*v*/*v*) NP-40, protease inhibitor cocktail (P8340, Sigma), and 1 mM PMSF). The lysates were centrifuged at 16,100 *g* at 4°C for 15 minutes. The supernatants were incubated with nProtein A Sepharose 4 Fast Flow (GE Healthcare) pre-bound with Iqcg antibody 4°C overnight with gentle rotation. The beads were washed four times with lysis buffer and prepared with SDS-PAGE sample buffer for Western blot analysis.

### Observation of the movements of spermatozoa and other cilia

For observation of the sperm movement, cauda epididymis of male mice was dissociated, cut into small pieces in PBS and kept in 37°C for 25 minutes to allow the spermatozoa to swim out. The collected spermatozoa were then transferred into a new culture dish and observed using an inverted phase contrast microscope (Nikon ECLIPSE Ti) equipped with a color digital camera and a video recording system.

For observation of the movements of cilia in trachea and oviduct, tissues were isolated in Dulbecco modified Eagle medium (DMEM) preheated in 37°C, cut into small pieces and observed in the same way as above.

### Statistical analysis

Results were expressed as means ± SEM. GraphPad Prism software was used for statistical analysis. Student's *t* test was used for determining the significance of the results. *P*<0.05 was considered statistically significant.

## Supporting Information

Figure S1 **Characterization of the specificity of Iqcg antibody.** (A) In Western blot analysis, this antibody specifically recognized a 55 kD band in the WT testis protein sample, which did not appear in the *Iqcg* KO testis sample. (B) This antibody specifically recognized Iqcg in WT testis section in immunohistochemical assay, whereas in the KO testis section, only very low background staining could be seen. (C) Iqcg antibody specificity in immunofluorescence assay. In the WT spermatozoa, the signal of the antibody was enriched in the flagella and the post-acrosomal region (middle panel). Such specific signal disappeared in the KO spermatozoa (left panel) or when the antibody was pre-blocked with excess antigen fragment (right panel).
(TIF)

Figure S2 **Detection of the potential truncated form of** ***Iqcg*** **in the KO mice.** *Iqcg* 3′primers were designed in the exon 7–9, which was after the trapping cassette. Two independent pairs of primers, *Iqcg* 5′-1 and *Iqcg* 5′-2, were designed in the 5′ region of *Iqcg* before the trapping cassette to detect the potential truncated form of *Iqcg* mRNA. PCR was performed using the WT or KO testis cDNA. The obtained DNA fragments were sequenced, and confirmed to be amplified from *Iqcg*, demonstrating the existence of a truncated form at mRNA level. Products of reverse transcription reaction with no mRNA templates were used as negative control (NC) of PCR.
(TIF)

Figure S3 **Histology of tissues in the WT and** ***Iqcg*** **KO mice.** For brain, scale bar  = 2 µm. For others, scale bar  = 50 µm.
(TIF)

Figure S4 **Histology of WT and** ***Iqcg*** **KO testis before spermiogenesis.** Scale bar  = 50 µm.
(TIF)

Figure S5 **Nuclear shaping defects of** ***Iqcg*** **KO spermatozoa displayed by DAPI staining.** Arrows indicate typical deformed nuclei. Scale bar  = 20 µm.
(TIF)

Figure S6 **Sperm flagellum formation was already disrupted in the developing spermatids.** Acetylated α-tubulin (green) was used as a marker for flagellum axoneme. In the KO spermatids, the axoneme was very short and sometimes abnormally frizzled. Imaged were merged from the layers of FITC, DAPI and DIC. Scale bar  = 20 µm.c
(TIF)

Figure S7 **Microtubules and actin filaments in WT and KO spermatids.** Immunofluorescence was performed on the drying down preparations corresponding with spermatids of different steps. Cells were double stained with α-tubulin (green) and β-actin (red) antibodies. DAPI was used to stain nucleus. Scale bar  = 5 µm.
(TIF)

Figure S8 **Immunohistochemical analysis of Iqcg in trachea and oviduct.** Iqcg was localized to the cilia both in trachea and cilia. Scale bar  = 20 µm.
(TIF)

Figure S9 **Histological and ultrastructural analyses of WT and** ***Iqcg*** **KO cilia in the trachea and oviduct.** Both the quantities or the lengths of the cilia in the *Iqcg* KO trachea and oviduct revealed no significant differences to the WT controls. The “9+2” pattern of microtubular arrangement also appeared intact. For H&E staining results, scale bars  = 20 µm. For TEM results, scale bars  = 500 nm.
(TIF)

Table S1 ***Iqcg*** **KO mice showed normal testis/body weight.**
(DOC)

Movie S1 **Motility of WT spermatozoa.**
(MP4)

Movie S2 **Motility of** ***Iqcg*** **KO spermatozoa.**
(MP4)

Movie S3 **Motility of cilia in WT trachea.**
(MP4)

Movie S4 **Motility of cilia in** ***Iqcg*** **KO trachea.**
(MP4)

Movie S5 **Motility of cilia in WT oviduct.**
(MP4)

Movie S6 **Motility of cilia in** ***Iqcg*** **KO oviduct.**
(MP4)

## References

[1] Cooke HJ, Saunders PTK (2002) Mouse models of male infertility. Nature Reviews Genetics 3: 790–801. 10.1038/nrg911 12360237

[2] Clermont Y (1972) Kinetics of spermatogenesis in mammals: seminiferous epithelium cycle and spermatogonial renewal. Physiol Rev 52: 198–236. 4621362 10.1152/physrev.1972.52.1.198

[3] Hess RA, Renato de Franca L (2008) Spermatogenesis and cycle of the seminiferous epithelium. Adv Exp Med Biol 636: 1–15. 19856159 10.1007/978-0-387-09597-4_1

[4] Ahmed EA, Rooij DG (2009) Staging of Mouse Seminiferous Tubule Cross-Sections. 558: 263–277. 10.1007/978-1-60761-103-5_16 19685330

[5] Yan W (2009) Male infertility caused by spermiogenic defects: Lessons from gene knockouts. Molecular and Cellular Endocrinology 306: 24–32. 19481682 10.1016/j.mce.2009.03.003 PMC5438260

[6] Mendoza-Lujambio I, Burfeind P, Dixkens C, Meinhardt A, Hoyer-Fender S, et al (2002) The Hook1 gene is non-functional in the abnormal spermatozoon head shape (azh) mutant mouse. Hum Mol Genet 11: 1647–1658. 12075009 10.1093/hmg/11.14.1647

[7] Cho C, Willis WD, Goulding EH, Jung-Ha H, Choi YC, et al (2001) Haploinsufficiency of protamine-1 or -2 causes infertility in mice. Nat Genet 28: 82–86. 11326282 10.1038/ng0501-82

[8] Cho C, Jung-Ha H, Willis WD, Goulding EH, Stein P, et al (2003) Protamine 2 deficiency leads to sperm DNA damage and embryo death in mice. Biol Reprod 69: 211–217. 12620939 10.1095/biolreprod.102.015115

[9] Yu YE, Zhang Y, Unni E, Shirley CR, Deng JM, et al (2000) Abnormal spermatogenesis and reduced fertility in transition nuclear protein 1-deficient mice. Proc Natl Acad Sci U S A 97: 4683–4688. 10781074 10.1073/pnas.97.9.4683 PMC18293

[10] Zhao M, Shirley CR, Hayashi S, Marcon L, Mohapatra B, et al (2004) Transition nuclear proteins are required for normal chromatin condensation and functional sperm development. Genesis 38: 200–213. 15083521 10.1002/gene.20019

[11] Kang-Decker N, Mantchev GT, Juneja SC, McNiven MA, van Deursen JM (2001) Lack of acrosome formation in Hrb-deficient mice. Science 294: 1531–1533. 11711676 10.1126/science.1063665

[12] Yao R, Ito C, Natsume Y, Sugitani Y, Yamanaka H, et al (2002) Lack of acrosome formation in mice lacking a Golgi protein, GOPC. Proceedings of the National Academy of Sciences 99: 11211–11216. 10.1073/pnas.162027899 PMC123235 12149515

[13] Escalier D, Silvius D, Xu X (2003) Spermatogenesis of mice lacking CK2alpha': failure of germ cell survival and characteristic modifications of the spermatid nucleus. Mol Reprod Dev 66: 190–201. 12950107 10.1002/mrd.10346

[14] Nishimura DY, Fath M, Mullins RF, Searby C, Andrews M, et al (2004) Bbs2-null mice have neurosensory deficits, a defect in social dominance, and retinopathy associated with mislocalization of rhodopsin. Proceedings of the National Academy of Sciences 101: 16588–16593. 10.1073/pnas.0405496101 PMC534519 15539463

[15] Tanaka H, Iguchi N, Toyama Y, Kitamura K, Takahashi T, et al (2004) Mice deficient in the axonemal protein Tektin-t exhibit male infertility and immotile-cilium syndrome due to impaired inner arm dynein function. Mol Cell Biol 24: 7958–7964. 15340058 10.1128/MCB.24.18.7958-7964.2004 PMC515054

[16] Miki K, Willis WD, Brown PR, Goulding EH, Fulcher KD, et al (2002) Targeted Disruption of the Akap4 Gene Causes Defects in Sperm Flagellum and Motility. Developmental Biology 248: 331–342. 12167408 10.1006/dbio.2002.0728

[17] Zhang Z, Shen X, Gude DR, Wilkinson BM, Justice MJ, et al (2009) MEIG1 is essential for spermiogenesis in mice. Proceedings of the National Academy of Sciences 106: 17055–17060. 10.1073/pnas.0906414106 PMC2746124 19805151

[18] Lorenzetti D, Bishop CE, Justice MJ (2004) Deletion of the Parkin coregulated gene causes male sterility in the quakingviable mouse mutant. Proceedings of the National Academy of Sciences 101: 8402–8407. 10.1073/pnas.0401832101 PMC420406 15148410

[19] Lehti MS, Kotaja N, Sironen A (2013) KIF3A is essential for sperm tail formation and manchette function. Molecular and Cellular Endocrinology 377: 44–55. 23831641 10.1016/j.mce.2013.06.030

[20] Pan Q, Zhu YJ, Gu BW, Cai X, Bai XT, et al (2008) A new fusion gene NUP98-IQCG identified in an acute T-lymphoid/myeloid leukemia with a t(3;11)(q29q13;p15)del(3)(q29) translocation. Oncogene 27: 3414–3423. 18084320 10.1038/sj.onc.1210999

[21] Harris TP, Schimenti KJ, Munroe RJ, Schimenti JC (2013) IQ Motif-Containing G (Iqcg) Is Required for Mouse Spermiogenesis. G3 (Bethesda). 10.1534/g3.113.009563 PMC3931569 24362311

[22] Borg CL, Wolski KM, Gibbs GM, O'Bryan MK (2009) Phenotyping male infertility in the mouse: how to get the most out of a 'non-performer'. Human Reproduction Update 16: 205–224. 19758979 10.1093/humupd/dmp032 PMC2816191

[23] Cao W, Gerton GL, Moss SB (2006) Proteomic profiling of accessory structures from the mouse sperm flagellum. Mol Cell Proteomics 5: 801–810. 16452089 10.1074/mcp.M500322-MCP200

[24] Kierszenbaum AL (2001) Spermatid manchette: plugging proteins to zero into the sperm tail. Mol Reprod Dev 59: 347–349. 11468770 10.1002/mrd.1040

[25] Kierszenbaum AL (2002) Intramanchette transport (IMT): Managing the making of the spermatid head, centrosome, and tail. Molecular Reproduction and Development 63: 1–4. 12211054 10.1002/mrd.10179

[26] Wolosewick JJ, Bryan JH (1977) Ultrastructural characterization of the manchette microtubules in the seminiferous epithelium of the mouse. Am J Anat 150: 301–331. 920632 10.1002/aja.1001500206

[27] Testa G, Schaft J, van der Hoeven F, Glaser S, Anastassiadis K, et al (2004) A reliable lacZ expression reporter cassette for multipurpose, knockout-first alleles. Genesis 38: 151–158. 15048813 10.1002/gene.20012

[28] Kierszenbaum AL, Rivkin E, Tres LL (2011) Cytoskeletal track selection during cargo transport in spermatids is relevant to male fertility. Spermatogenesis 1: 221–230. 22319670 10.4161/spmg.1.3.18018 PMC3271664

[29] Sperry AO (2012) The dynamic cytoskeleton of the developing male germ cell. Biol Cell 104: 297–305. 22276751 10.1111/boc.201100102 PMC3845902

[30] Kierszenbaum AL, Rivkin E, Tres LL, Yoder BK, Haycraft CJ, et al (2011) GMAP210 and IFT88 are present in the spermatid golgi apparatus and participate in the development of the acrosome-acroplaxome complex, head-tail coupling apparatus and tail. Developmental Dynamics 240: 723–736. 21337470 10.1002/dvdy.22563 PMC4175411

[31] Zhou J, Du YR, Qin WH, Hu YG, Huang YN, et al (2008) RIM-BP3 is a manchette-associated protein essential for spermiogenesis. Development 136: 373–382. 19091768 10.1242/dev.030858

[32] Kierszenbaum AL, Rivkin E, Tres LL (2003) The actin-based motor myosin Va is a component of the acroplaxome, an acrosome-nuclear envelope junctional plate, and of manchette-associated vesicles. Cytogenet Genome Res 103: 337–344. 15051957 10.1159/000076822

[33] Li Z, Sacks DB (2003) Elucidation of the interaction of calmodulin with the IQ motifs of IQGAP1. J Biol Chem 278: 4347–4352. 12446675 10.1074/jbc.M208579200

[34] Oh EC, Katsanis N (2012) Cilia in vertebrate development and disease. Development 139: 443–448. 22223675 10.1242/dev.050054 PMC3275291

[35] Garcia-Gonzalo FR, Reiter JF (2012) Scoring a backstage pass: Mechanisms of ciliogenesis and ciliary access. The Journal of Cell Biology 197: 697–709. 22689651 10.1083/jcb.201111146 PMC3373398

[36] Ishikawa H, Marshall WF (2011) Ciliogenesis: building the cell's antenna. Nature Reviews Molecular Cell Biology 12: 222–234. 21427764 10.1038/nrm3085

[37] Escalier D (2006) Knockout mouse models of sperm flagellum anomalies. Hum Reprod Update 12: 449–461. 16565154 10.1093/humupd/dml013

[38] Fath MA, Mullins RF, Searby C, Nishimura DY, Wei J, et al (2005) Mkks-null mice have a phenotype resembling Bardet-Biedl syndrome. Hum Mol Genet 14: 1109–1118. 15772095 10.1093/hmg/ddi123

[39] Zhou J, Yang F, Leu NA, Wang PJ (2012) MNS1 Is Essential for Spermiogenesis and Motile Ciliary Functions in Mice. PLoS Genet 8: e1002516. 22396656 10.1371/journal.pgen.1002516 PMC3291534

[40] Gherman A, Davis EE, Katsanis N (2006) The ciliary proteome database: an integrated community resource for the genetic and functional dissection of cilia. Nat Genet 38: 961–962. 16940995 10.1038/ng0906-961

[41] Darszon A, Nishigaki T, Beltran C, Trevino CL (2011) Calcium channels in the development, maturation, and function of spermatozoa. Physiol Rev 91: 1305–1355. 22013213 10.1152/physrev.00028.2010

[42] Berrios J, Osses N, Opazo C, Arenas G, Mercado L, et al (1998) Intracellular Ca2+ homeostasis in rat round spermatids. Biol Cell 90: 391–398. 9835013

[43] Slaughter GR, Needleman DS, Means AR (1987) Developmental regulation of calmodulin, actin, and tubulin RNAs during rat testis differentiation. Biol Reprod 37: 1259–1270. 2450594 10.1095/biolreprod37.5.1259

[44] Slaughter GR, Meistrich ML, Means AR (1989) Expression of RNAs for calmodulin, actins, and tubulins in rat testis cells. Biol Reprod 40: 395–405. 2720034 10.1095/biolreprod40.2.395

[45] Sano M, Ohshima AS, Kawamura N, Kitajima S, Mizutani A (1987) Immunohistochemical study of calmodulin in developing mouse testis. J Exp Zool 241: 51–59. 3549967 10.1002/jez.1402410107

[46] Jones HP, Lenz RW, Palevitz BA, Cormier MJ (1980) Calmodulin localization in mammalian spermatozoa. Proc Natl Acad Sci U S A 77: 2772–2776. 6994106 10.1073/pnas.77.5.2772 PMC349486

[47] Weinman S, Ores-Carton C, Escaig F, Feinberg J, Puszkin S (1986) Calmodulin immunoelectron microscopy: redistribution during ram spermatogenesis and epididymal maturation. II. Journal of Histochemistry & Cytochemistry 34: 1181–1193. 3734420 10.1177/34.9.3734420

[48] Kann ML, Feinberg J, Rainteau D, Dadoune JP, Weinman S, et al (1991) Localization of calmodulin in perinuclear structures of spermatids and spermatozoa: a comparison of six mammalian species. Anat Rec 230: 481–488. 1928753 10.1002/ar.1092300407

[49] Means AR, Tash JS, Chafouleas JG, Lagace L, Guerriero V (1982) Regulation of the cytoskeleton by Ca2+-calmodulin and cAMP. Ann N Y Acad Sci 383: 69–84. 6283996 10.1111/j.1749-6632.1982.tb23162.x

[50] Adelstein RS (1982) Calmodulin and the regulation of the actin-myosin interaction in smooth muscle and nonmuscle cells. Cell 30: 349–350. 6128074 10.1016/0092-8674(82)90232-x

[51] Keith CH, Bajer AS, Ratan R, Maxfield FR, Shelanski ML (1986) Calcium and calmodulin in the regulation of the microtubular cytoskeleton. Ann N Y Acad Sci 466: 375–391. 3524372 10.1111/j.1749-6632.1986.tb38407.x

[52] Hammer JA, Sellers JR (2011) Walking to work: roles for class V myosins as cargo transporters. Nature Reviews Molecular Cell Biology. 10.1038/nrm3248 22146746

[53] Baccetti B, Burrini AG, Capitani S, Collodel G, Moretti E, et al (1993) Notulae seminologicae. 2. The 'short tail' and 'stump' defect in human spermatozoa. Andrologia 25: 331–335. 8279704

[54] Baccetti B, Capitani S, Collodel G, Di Cairano G, Gambera L, et al (2001) Genetic sperm defects and consanguinity. Hum Reprod 16: 1365–1371. 11425814 10.1093/humrep/16.7.1365

[55] Kotaja N, Kimmins S, Brancorsini S, Hentsch D, Vonesch JL, et al (2004) Preparation, isolation and characterization of stage-specific spermatogenic cells for cellular and molecular analysis. Nat Methods 1: 249–254. 16144087 10.1038/nmeth1204-249

