# Supplementary figures and images for: Iqcg Is Essential for Sperm Flagellum Formation in Mice
Source: PLoS One. 2014 May 21;9(5):e98053. doi: 10.1371/journal.pone.0098053 (PMC4029791; doi:10.1371/journal.pone.0098053)

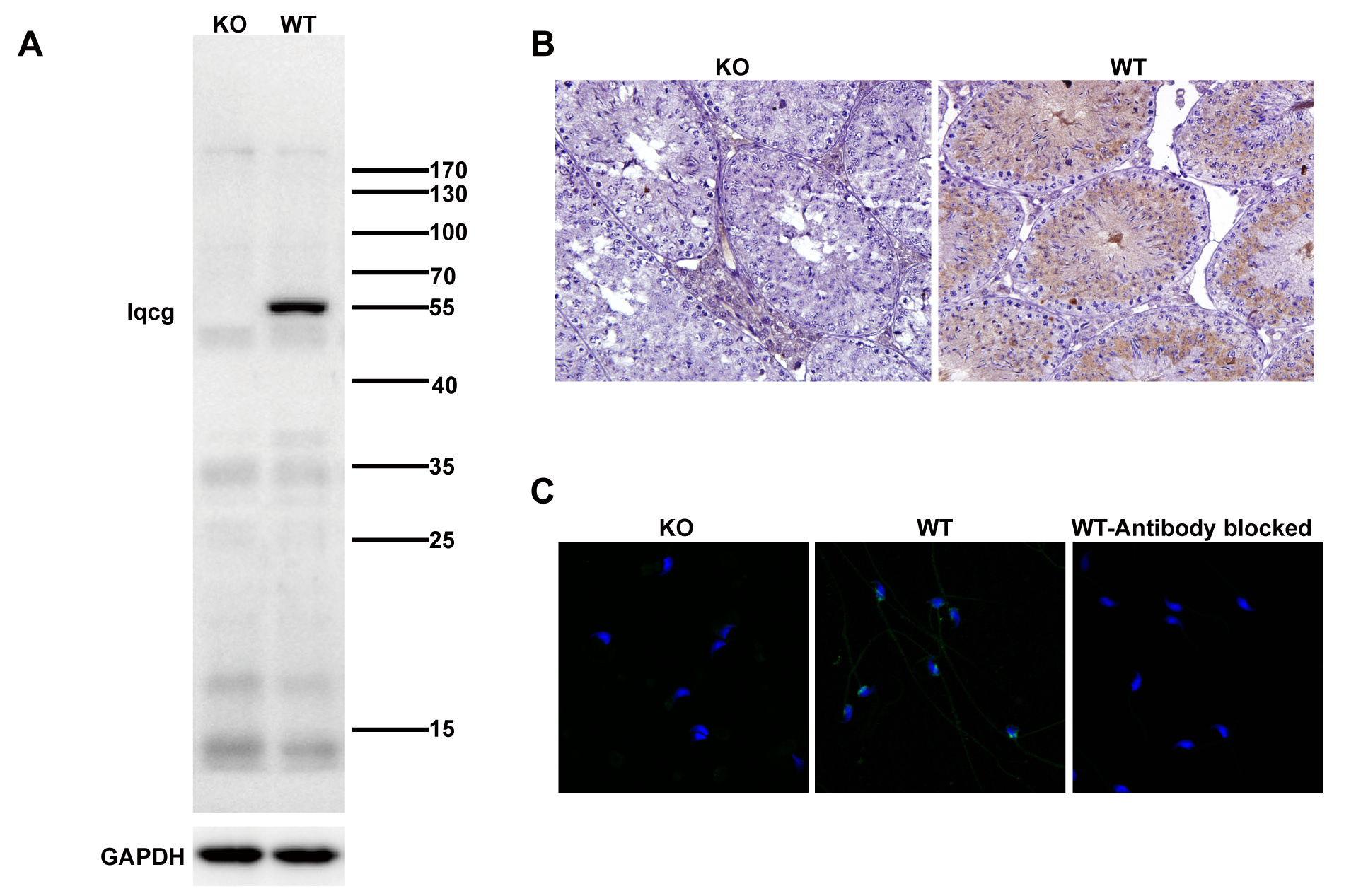

Supplement: Figure S1 — Characterization of the specificity of Iqcg antibody. (A) In Western blot analysis, this antibody specifically recognized a 55 kD band in the WT testis protein sample, which did not appear in the Iqcg KO testis sample. (B) This antibody specifically recognized Iqcg in WT testis section in immunohistochemical assay, whereas in the KO testis section, only very low background staining could be seen. (C) Iqcg antibody specificity in immunofluorescence assay. In the WT spermatozoa, the signal of the antibody was enriched in the flagella and the post-acrosomal region (middle panel). Such specific signal disappeared in the KO spermatozoa (left panel) or when the antibody was pre-blocked with excess antigen fragment (right panel). (TIF) [file pone.0098053.s001.tif]

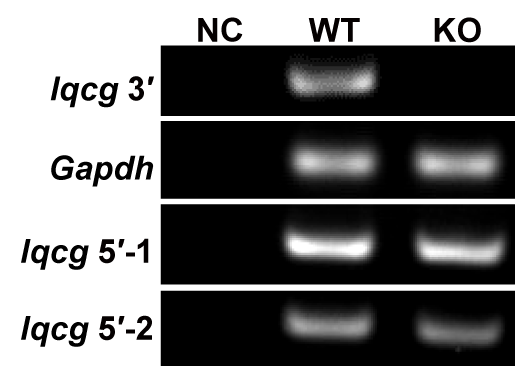

Supplement: Figure S2 — Detection of the potential truncated form of Iqcg in the KO mice. Iqcg 3′primers were designed in the exon 7–9, which was after the trapping cassette. Two independent pairs of primers, Iqcg 5′-1 and Iqcg 5′-2, were designed in the 5′ region of Iqcg before the trapping cassette to detect the potential truncated form of Iqcg mRNA. PCR was performed using the WT or KO testis cDNA. The obtained DNA fragments were sequenced, and confirmed to be amplified from Iqcg, demonstrating the existence of a truncated form at mRNA level. Products of reverse transcription reaction with no mRNA templates were used as negative control (NC) of PCR. (TIF) [file pone.0098053.s002.tif]

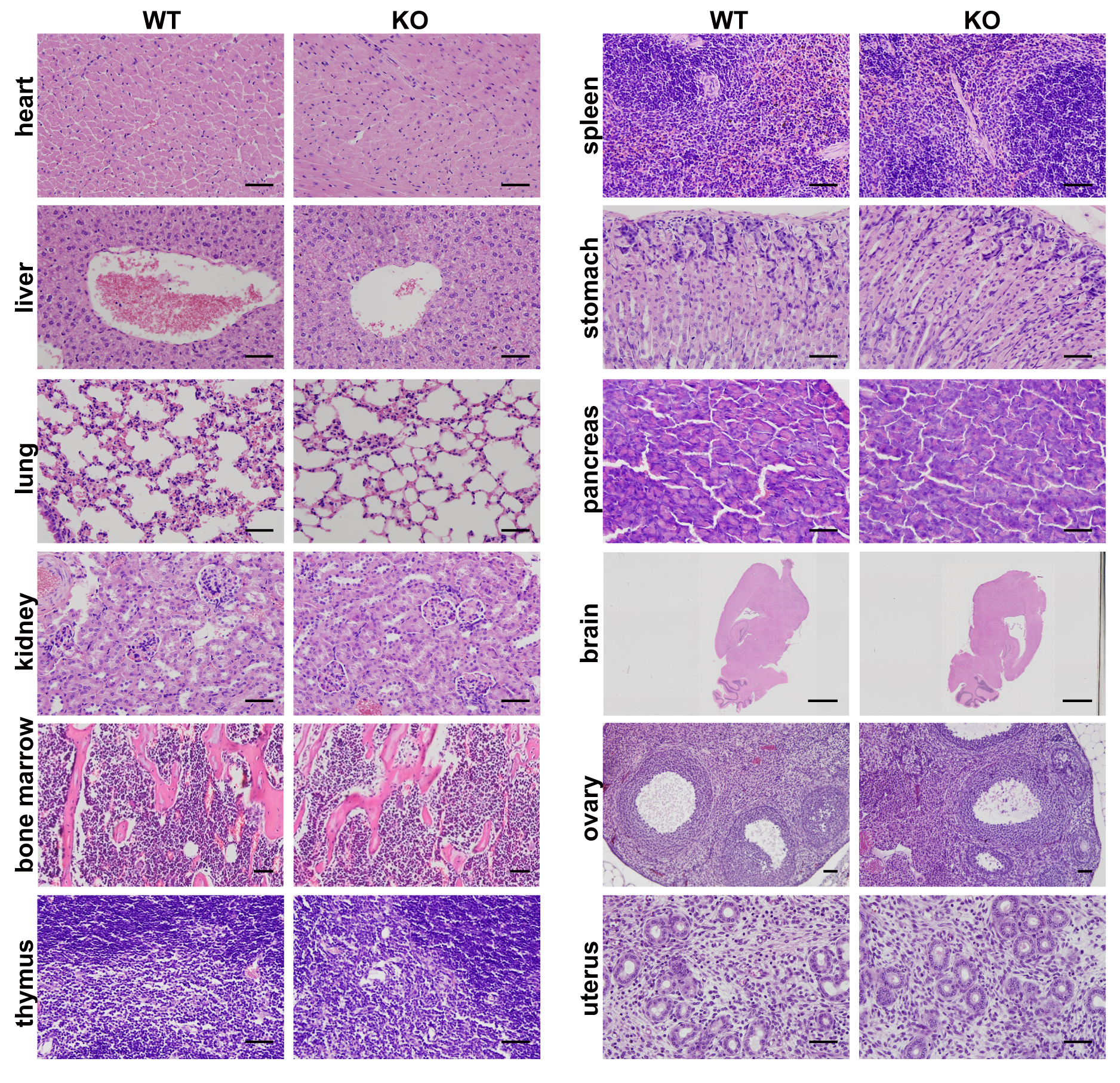

Supplement: Figure S3 — Histology of tissues in the WT and Iqcg KO mice. For brain, scale bar = 2 µm. For others, scale bar = 50 µm. (TIF) [file pone.0098053.s003.tif]

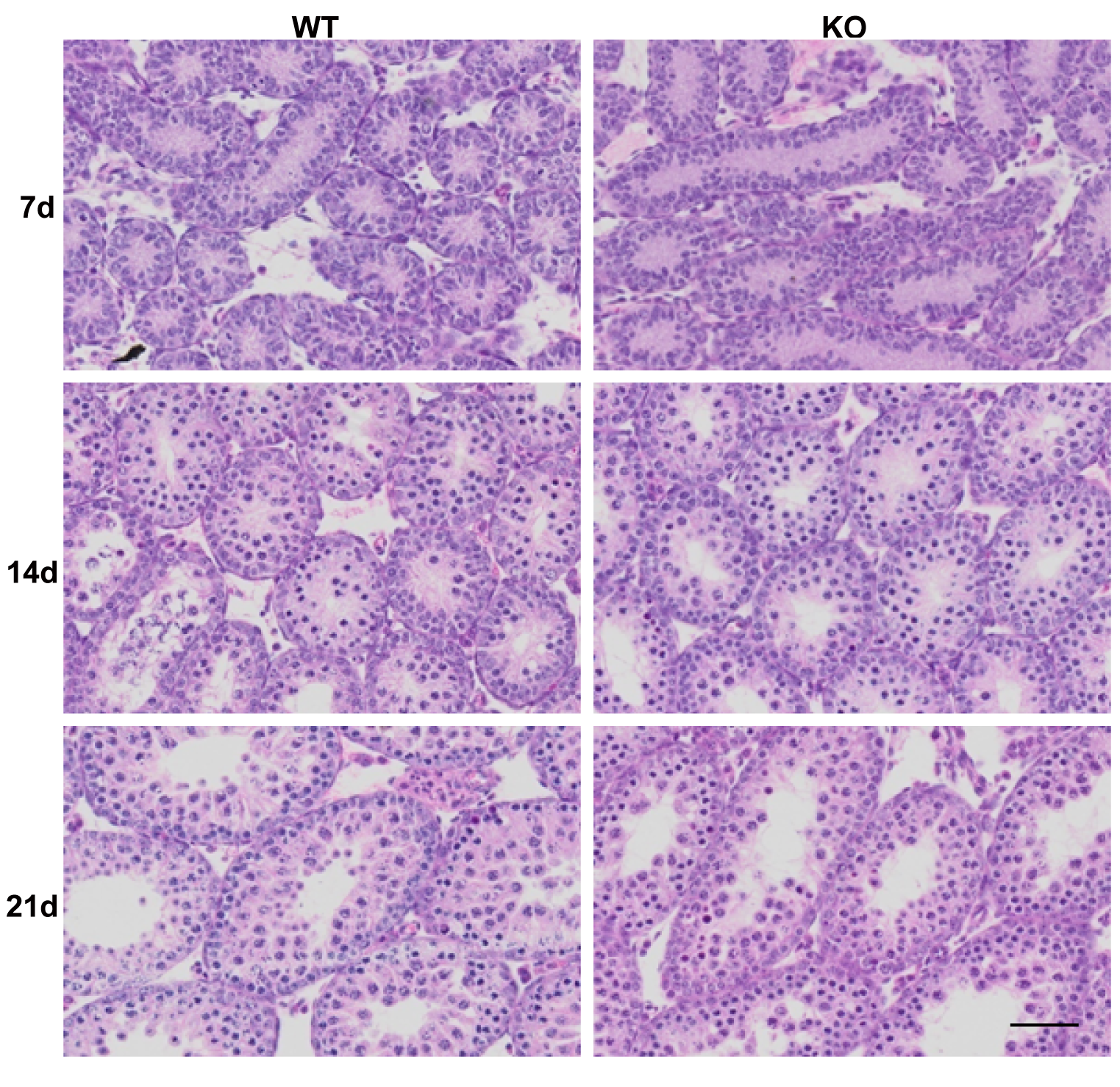

Supplement: Figure S4 — Histology of WT and Iqcg KO testis before spermiogenesis. Scale bar = 50 µm. (TIF) [file pone.0098053.s004.tif]

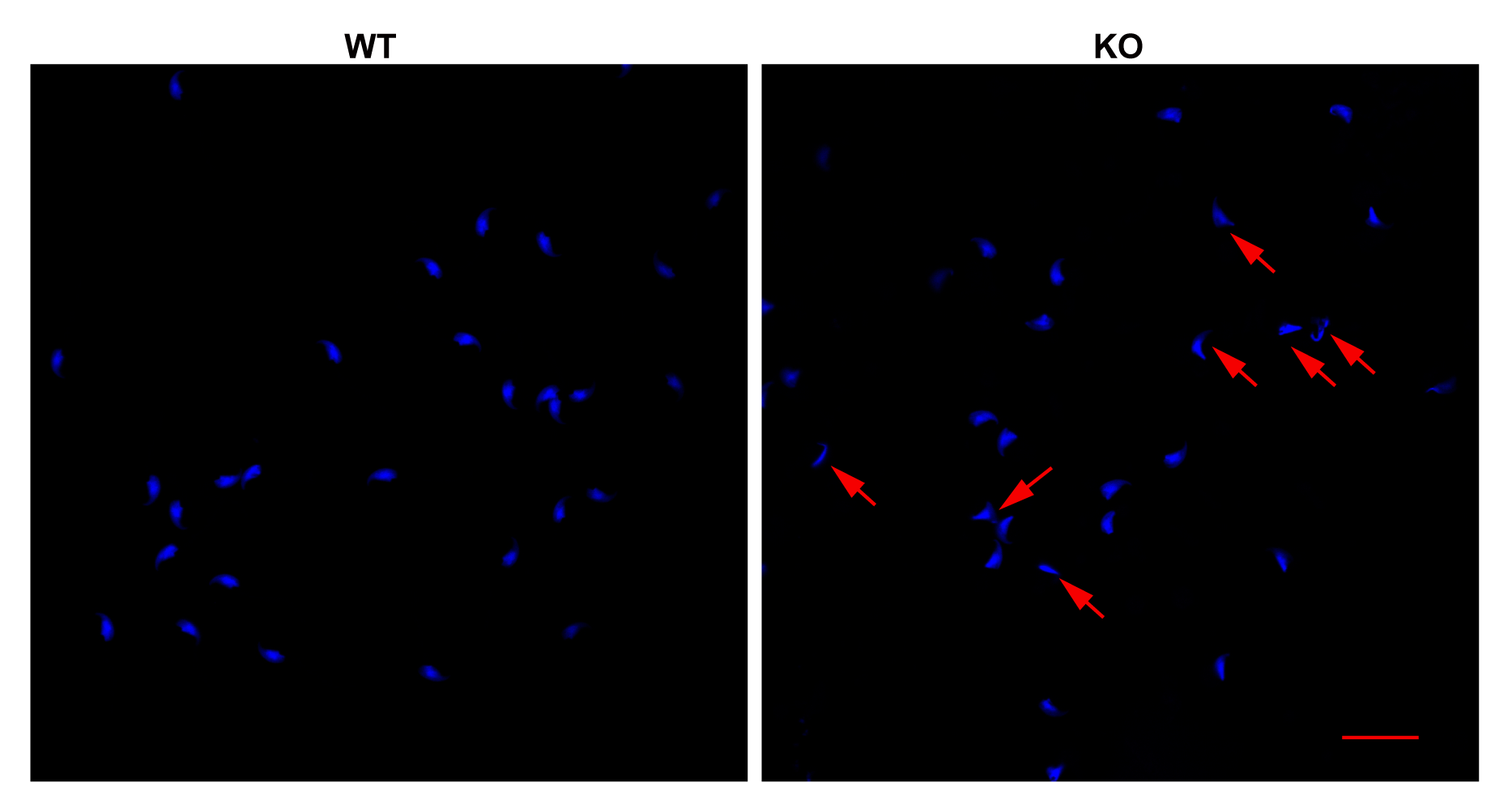

Supplement: Figure S5 — Nuclear shaping defects of Iqcg KO spermatozoa displayed by DAPI staining. Arrows indicate typical deformed nuclei. Scale bar = 20 µm. (TIF) [file pone.0098053.s005.tif]

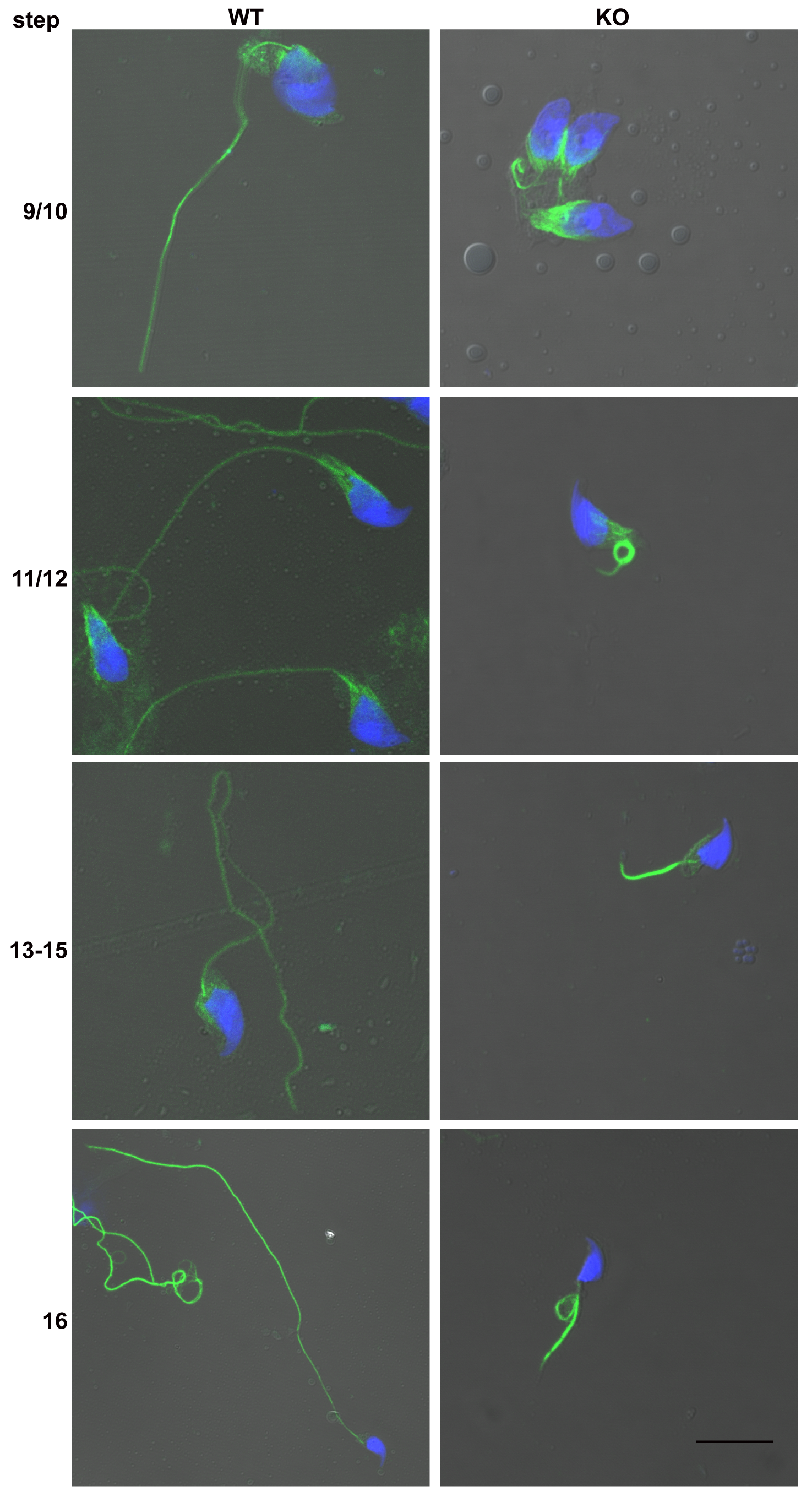

Supplement: Figure S6 — Sperm flagellum formation was already disrupted in the developing spermatids. Acetylated α-tubulin (green) was used as a marker for flagellum axoneme. In the KO spermatids, the axoneme was very short and sometimes abnormally frizzled. Imaged were merged from the layers of FITC, DAPI and DIC. Scale bar = 20 µm.c (TIF) [file pone.0098053.s006.tif]

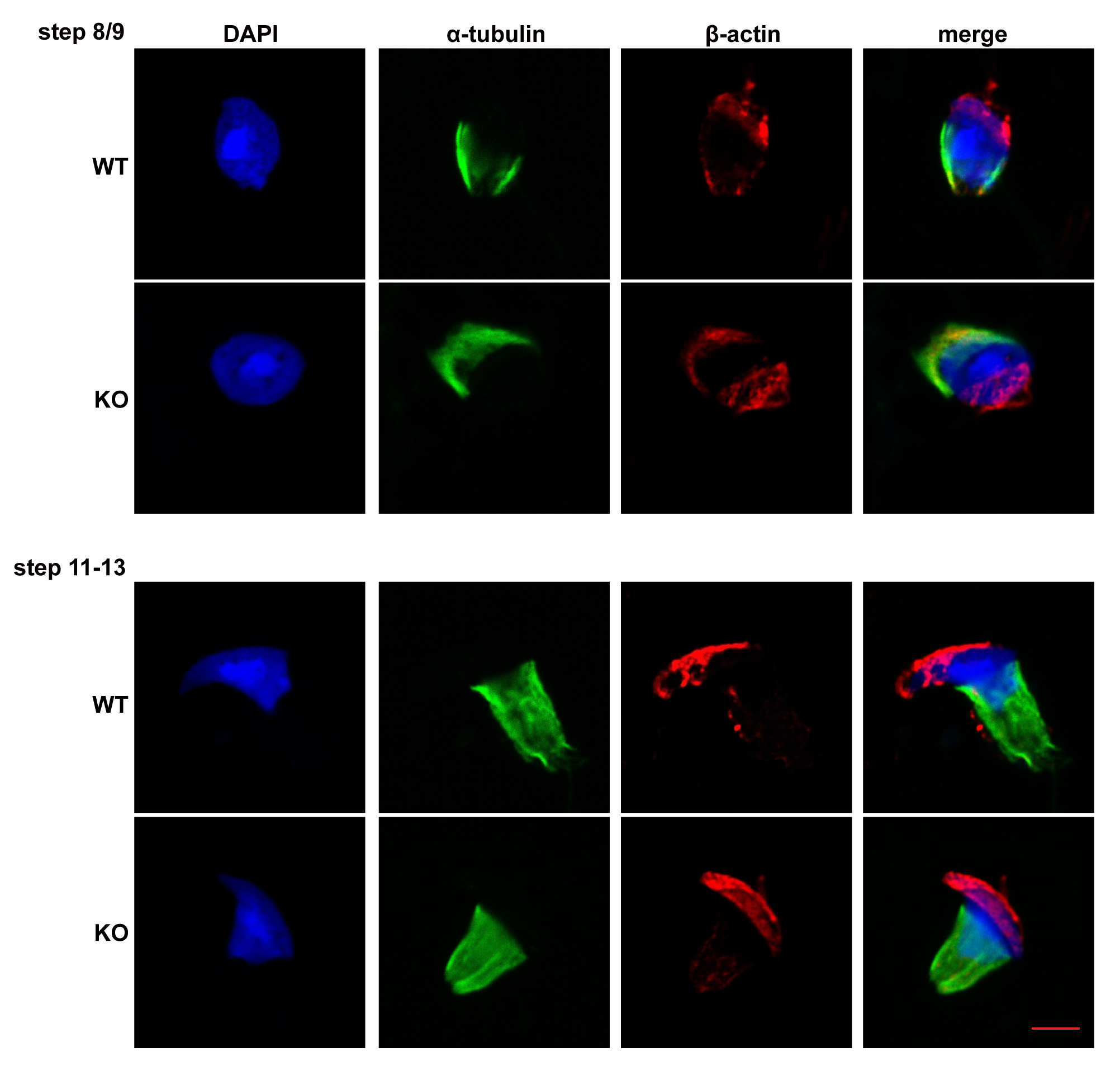

Supplement: Figure S7 — Microtubules and actin filaments in WT and KO spermatids. Immunofluorescence was performed on the drying down preparations corresponding with spermatids of different steps. Cells were double stained with α-tubulin (green) and β-actin (red) antibodies. DAPI was used to stain nucleus. Scale bar = 5 µm. (TIF) [file pone.0098053.s007.tif]

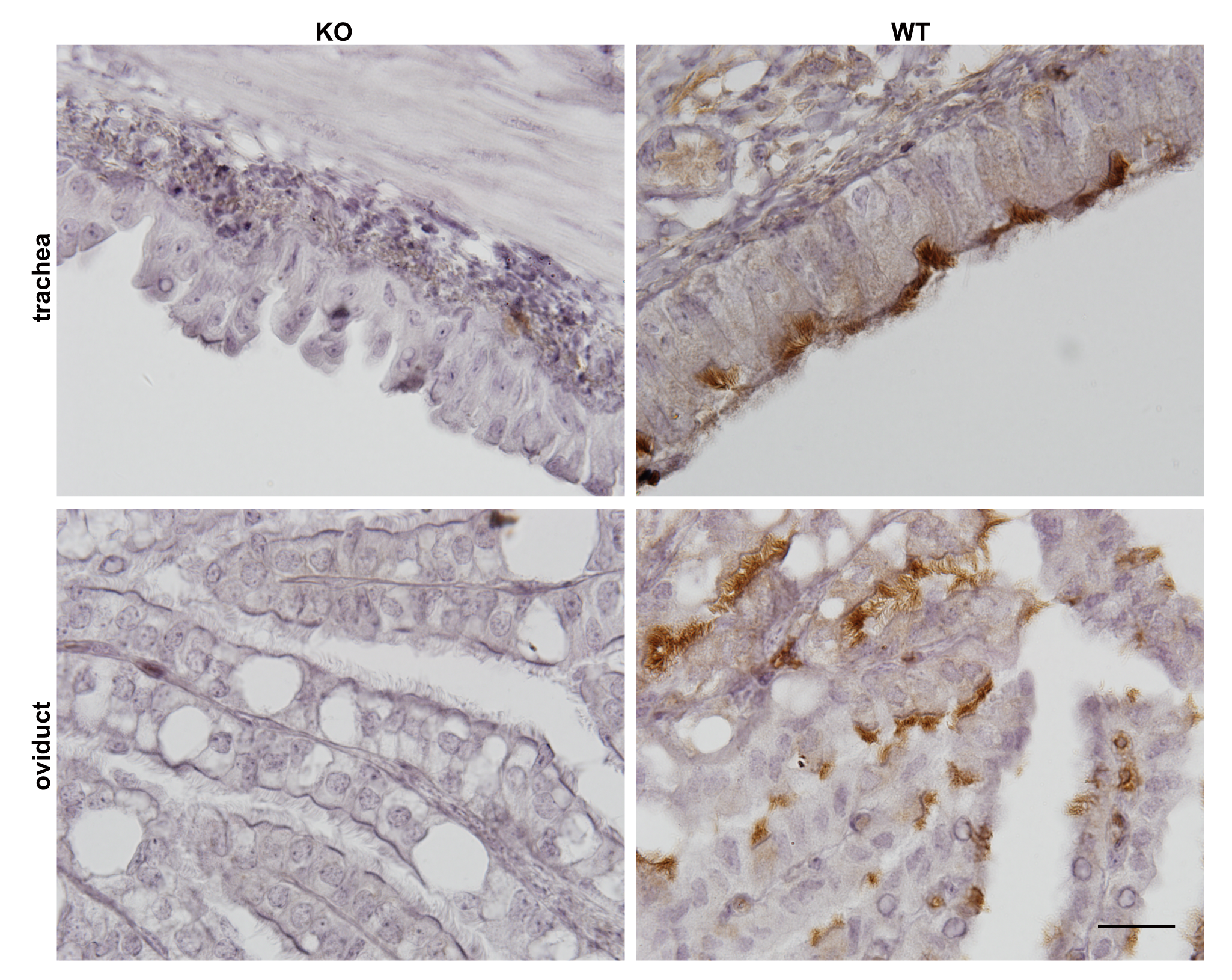

Supplement: Figure S8 — Immunohistochemical analysis of Iqcg in trachea and oviduct. Iqcg was localized to the cilia both in trachea and cilia. Scale bar = 20 µm. (TIF) [file pone.0098053.s008.tif]

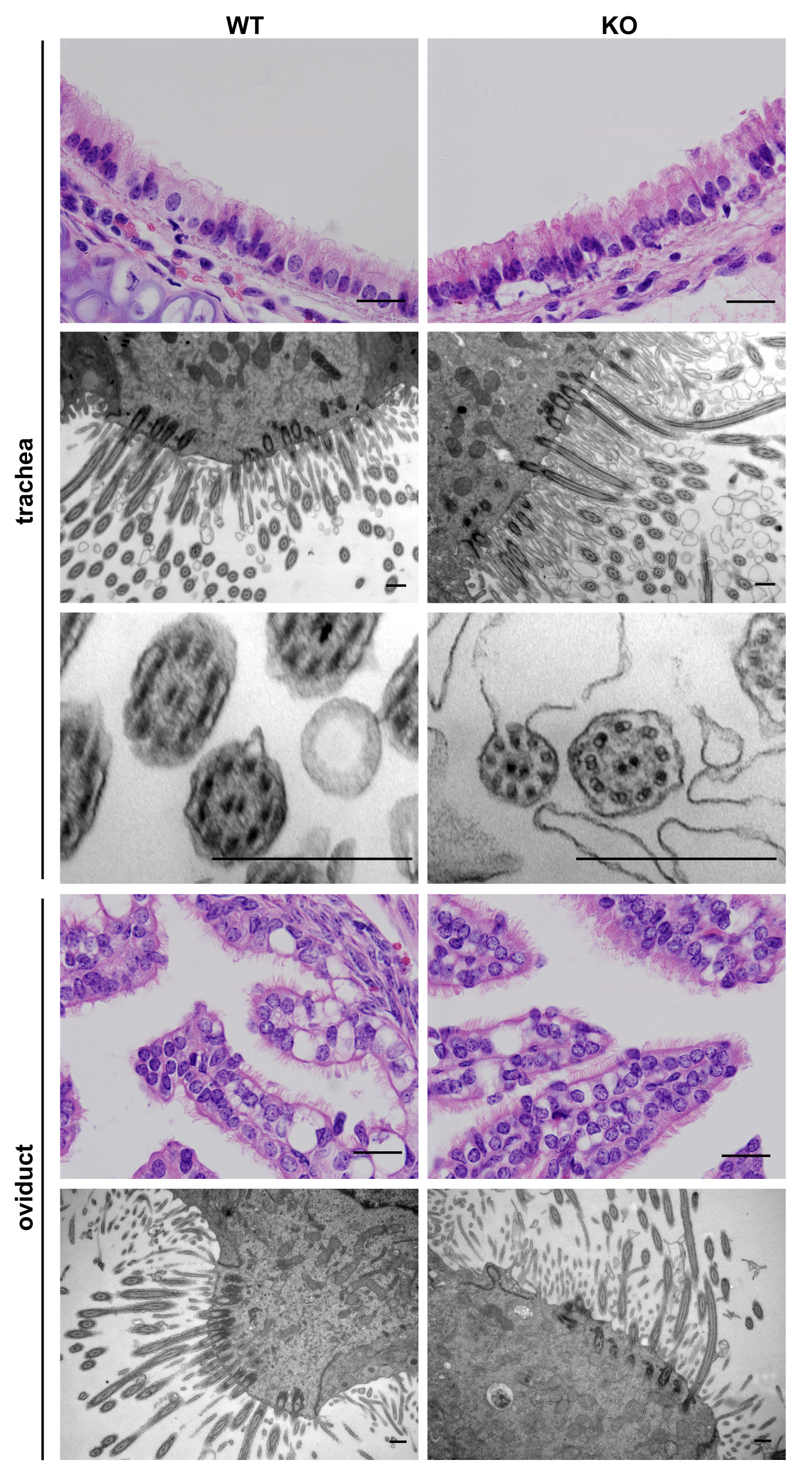

Supplement: Figure S9 — Histological and ultrastructural analyses of WT and Iqcg KO cilia in the trachea and oviduct. Both the quantities or the lengths of the cilia in the Iqcg KO trachea and oviduct revealed no significant differences to the WT controls. The “9+2” pattern of microtubular arrangement also appeared intact. For H&E staining results, scale bars = 20 µm. For TEM results, scale bars = 500 nm. (TIF) [file pone.0098053.s009.tif]
