# Supplementary material for: Iqcg Is Essential for Sperm Flagellum Formation in Mice
Source: PLoS One. 2014 May 21;9(5):e98053. doi: 10.1371/journal.pone.0098053 (PMC4029791; doi:10.1371/journal.pone.0098053)
Supplement: Table S1 — Iqcg KO mice showed normal testis/body weight. (DOC) [file pone.0098053.s010.doc]

**Table S1. *Iqcg* KO mice showed normal testis/body weight**

|  | **WT** | **KO** |
| --- | --- | --- |
| **Testis weight(mg)** | 90.6±2.7 | 87.2±3.9 |
| **Body weight(g)** | 27.8±0.8 | 25.7±0.7 |
| **Testis/body(×10^-3^)** | 3.27±0.10 | 3.58±0.23 |
| *n* = 12 each for WT and KO mice. Values are presented as means ± SEM. | | |
